# Inducing Single-Handed Helicity in a Twisted Molecular Nanoribbon

**DOI:** 10.1021/jacs.1c12385

**Published:** 2022-01-31

**Authors:** Rajeev K. Dubey, Manuel Melle-Franco, Aurelio Mateo-Alonso

**Affiliations:** † POLYMAT, University of the Basque Country UPV/EHU, Avenida Tolosa 72, 20018 Donostia-San Sebastian, Spain; ‡ CICECO, Aveiro Institute of Materials, Department of Chemistry, University of Aveiro, 3810-193 Aveiro, Portugal; § Ikerbasque, Basque Foundation for Science, 48009 Bilbao, Spain

## Abstract

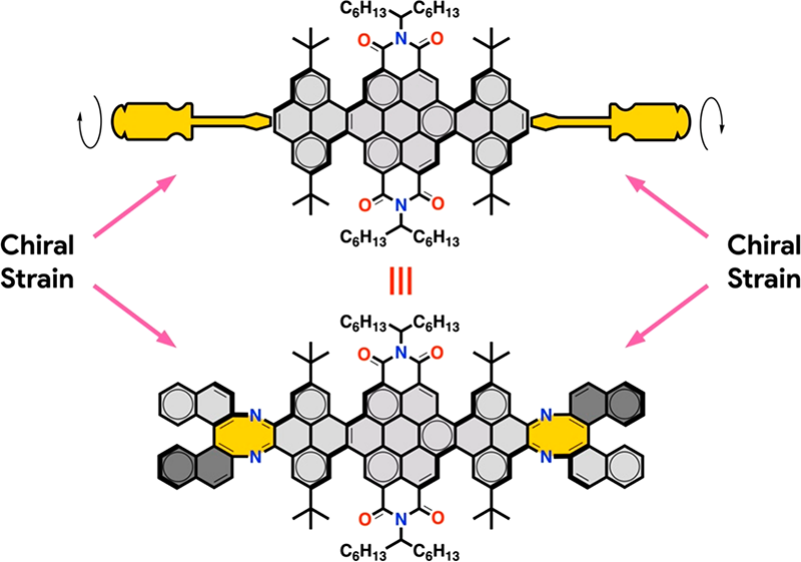

Molecular conformation has an important role in chemistry and materials science. Molecular nanoribbons can adopt chiral twisted helical conformations. However, the synthesis of single-handed helically twisted molecular nanoribbons still represents a considerable challenge. Herein, we describe an asymmetric approach to induce single-handed helicity with an excellent degree of conformational discrimination. The chiral induction is the result of the chiral strain generated by fusing two oversized chiral rings and of the propagation of that strain along the nanoribbon’s backbone.

## Introduction

Conformational isomerism is a fundamental concept in chemistry that provides an overall view of the different spatial arrangements that the atoms of a molecule can adopt. These arrangements define the shape(s) of a molecule and determine its properties. Molecular conformation is particularly relevant in the biological activity of natural and synthetic substances, since bio(macro)molecules, although very flexible, adopt specific conformations from which function evolves. Also, molecular conformation has an important role in chemistry and materials science, since different conformations may show different reactivities, self-organization behavior, interaction modes with other molecules, and physical properties.

**Figure 1:**
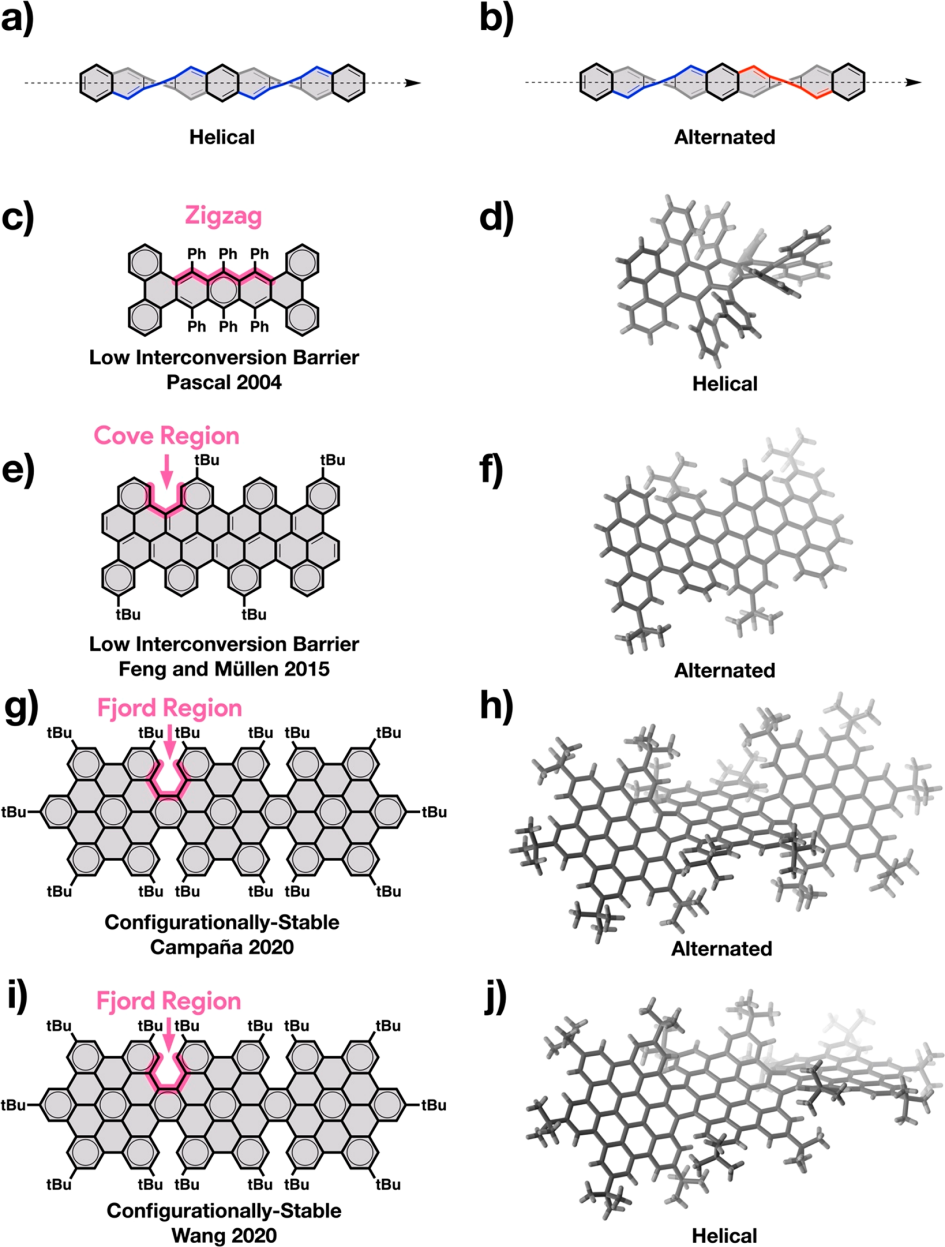
General structures of (a) helical and (b) alternated conformations of longitudinally twisted molecular NRs. Examples of twisted NR segments with (c) zigzag,^47^ (e) cove,^27^ and (g,i) fjord^42,43^ edges and their most stable (d) helical, (f) alternated, (h) alternated, and (j) helical conformations.

Nanographenes^1−8^—polycyclic aromatic hydrocarbons that extend over 1 nm—can adopt a broad range of nonplanar conformations^9−23^ that challenge the perception of aromatic systems as rigid and flat structures. Among these, molecular graphene nanoribbons (NRs),^2,24−55^—monodisperse 1D nanographenes—can adopt twisted conformations^10,27,31,33,41−43,46−52,56^ by introducing strain along the longitudinal edges of the NRs through steric crowding. In the case of NRs with more than two twists, helical (Figure 1a), alternated (Figure 1b), and mixed helical and alternated conformations can be obtained. An important aspect of helical conformations is their inherent chirality, which allows combining the chiroptical properties that emerge from homochiral molecules with the electronic, optical, and electrical properties that emerge from π-extended NRs. Homochiral helical NRs have a lot of potential in electronic and spintronic applications that exploit the absorption and emission of circularly polarized light^57−60^ and chiral induced spin selectivity.^57,61−63^

Despite the advances in the chemistry of molecular NRs,^2,24−55^ the synthesis of single-handed helically twisted molecular NRs still represents a considerable challenge. An important aspect is the configurational stability of the NRs, which depends on the NR’s edge and on the steric hindrance of the overcrowding groups. For example, the resolved helical enantiomers of overcrowded zigzag-edged tetrabenzopentacenes racemize in solution at ambient conditions (Figure 1c,d).^47^ Another example are cove-edged NRs that, although in a crystal structure they have shown to adopt an alternated conformation (Figure 1e,f),^27^ in solution, as the interconversion barriers are low, generally exist as a optically inactive mixtures of helical, alternated, and mixed conformers in constant exchange.^27,48,49,52^ Exceptionally, the interconversion barriers of NRs with fjord edges are sufficiently high to yield configurationally stable NRs (Figure 1g–j),^42,43,51^ some of which have been resolved by chiral high-performance liquid chromatography (HPLC).^42^ Asymmetric methods for the synthesis of single-handed twisted NRs, such as chiral induction or enantioselective synthesis, remain to be developed.

**Figure 2:**
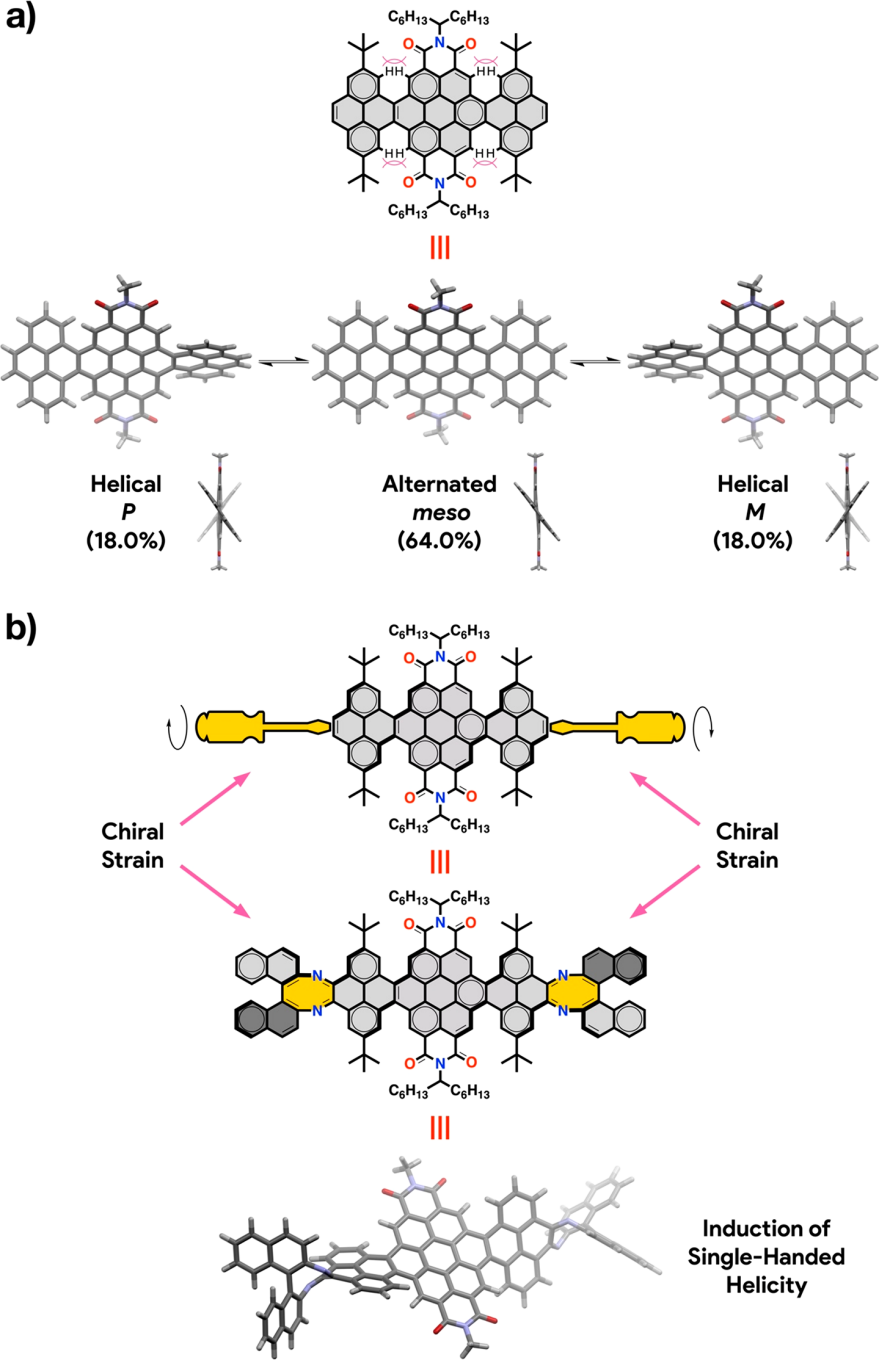
(a) Conformational isomers of **NR-7**. Percentual conformer populations at 25 °C are indicated between brackets. (b) Conceptual and experimental approach for the induction of single-handed helicity.

Herein we report an unprecedented approach to induce the preferential formation of single-handed helical conformations in a conformationally flexible molecular NR (Figure 2). This type of NRs are nonplanar because of the steric congestion generated by the inner hydrogen atoms at the cove regions. The four cove regions in the NRs produce an inseparable and optically inactive mixture of helical (*P* and *M*) and alternated conformers in constant exchange (Figure 2a) because of the low interconversion barrier. Our approach to induce single-handed helicity on such molecular NRs is based on the same principle used to twist a ribbon macroscopically, namely, the application of torque of the same sign at the ends of the ribbon (Figure 2b). To implement this principle at the molecular level, we have introduced two chiral 8-membered diazacyclooctatetraene rings by fusing enantiomerically pure 1,1′-binaphthyl-2,2′-diamine (BINAM) precursors at both ends of the aromatic framework. In this process, the axial chirality of BINAM is transferred to the newly formed 8-membered rings. The chiral strain generated by the oversized chiral 8-membered rings induces the formation of a helical conformer of the same handedness as the chiral strain with a high degree of conformational discrimination. Consequently, the chiral NRs show chiroptical properties that extend over the UV–vis up to 600 nm. Theoretical calculations reproduce the experimental findings and allow confirming that the chiral induction is a strain-induced process.

## Results and Discussion

### Synthesis and Characterization

**Scheme 1:**
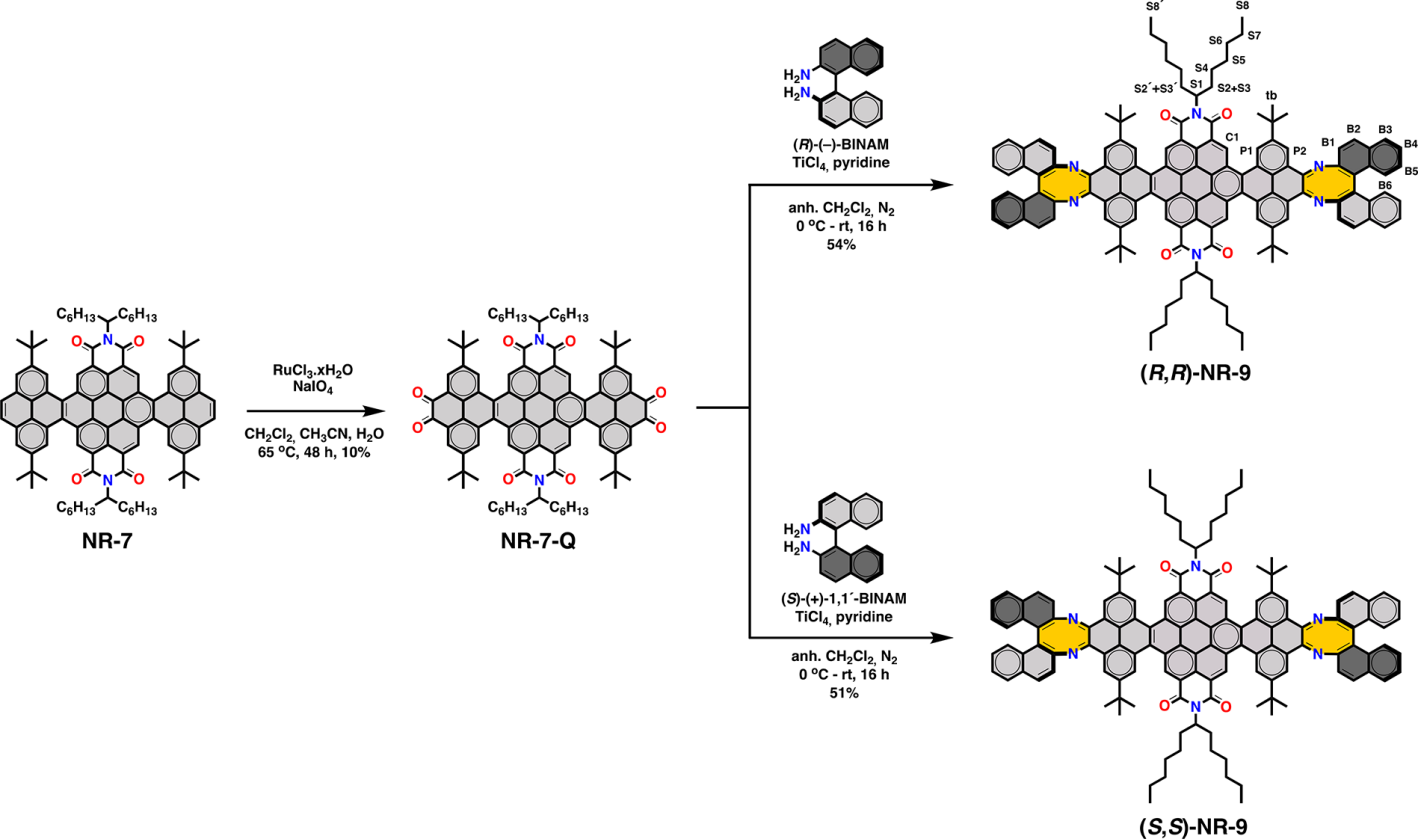
Synthesis of Homochiral Helical Nanoribbons (*R*,*R*)-**NR-9** and (*S*,*S*)-**NR-9**

The starting point for the synthesis of **NR-9** is compound **NR-7** (Scheme 1) that was obtained by the fusion of two pyrene chromophores to the bay regions of a perylene bisimide using Suzuki coupling and a Scholl-type intramolecular oxidative cyclodehydrogenation following a reported procedure.^46^ 1-Hexylheptyl and *tert*-butyl substituents were introduced respectively on the perylene and pyrene precursors to ensure the solubility of the intermediates and of the final NRs. Then, the K-regions of both pyrene residues of **NR-7** were oxidized to *o*-dione functionalities by NaIO_4_ catalyzed by RuCl_3_ yielding **NR-7-Q**.^46^ Cyclocondensation between **NR-7-Q** with either (*R*)-(+)- or (*S*)-(−)-BINAM were carried out in the presence TiCl_4_ at r.t. to yield respectively (*R*,*R*)-**NR-9** and (*S*,*S*)-**NR-9** as red solids in good yields after purification by column chromatography (∼50%).

**Figure 3:**
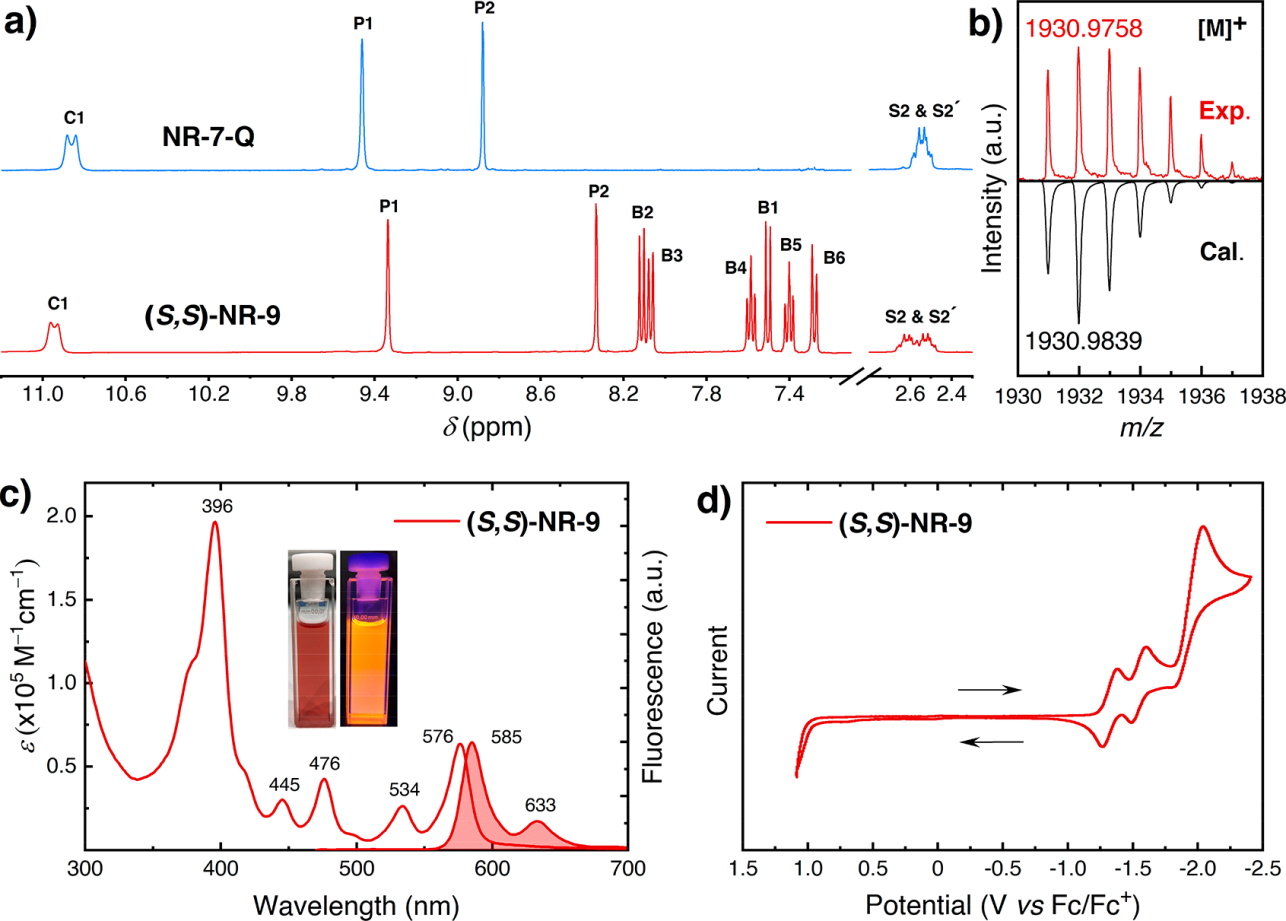
(a) A comparison of selected regions of ^1^H NMR spectra (CD_2_Cl_2_, 500 MHz) of **NR-7-Q** and (*S*,*S*)-**NR-9**. (b) HR-MS of (*S*,*S*)-**NR-9**. (c) UV–vis absorption and fluorescence (colored trace) spectra of (*S*,*S*)-**NR-9** (3.64 μM, λ_ex_ = 444 nm) in toluene. (d) Cyclic voltammogram (0.1 M *n*Bu_4_PF_6_ in CH_2_Cl_2_, scan rate = 50 mVs^–1^) of (*S*,*S*)-**NR-9**.

Despite their length (3.1 nm) and their large aromatic core constituted by 96 conjugated atoms (C_92_N_4_), molecular nanoribbons (*R*,*R*)-**NR-9** and (*S*,*S*)-**NR-9** are highly soluble (∼100 mg/mL) in a variety of organic solvents at room temperature, such as dichloromethane, chloroform, toluene, diethyl ether, THF, DMF, and NMP. The structure of (*R*,*R*)-**NR-9** and (*S*,*S*)-**NR-9** was unambiguously established by ^1^H and ^13^C NMR spectroscopy, and high-resolution mass spectrometry. The ^1^H NMR spectra of (*S*,*S*)-**NR-9** exhibited well-resolved proton signals that correspond to the binaphthyl, pyrene, and coronene residues. The signals corresponding to enantiotopic S2/S2′ proton couple located on the 1-hexylheptyl split into two individual signals (the lettering assignments are shown on Scheme 1), meanwhile in **NR-7** and **NR7-Q** the same proton couple resonate together (Figure 3a). This splitting indicates the presence of a chiral environment in the center of the longitudinal edges of aromatic core. The structure of **NR-9** was further confirmed by HR MALDI-TOF-MS spectra that showed the molecular ion peak (M^+^) and isotopic distributions consistent with the molecular weight (Figure 3b).

### Optoelectronic and Redox Properties

Solutions of **NR-9** show an intense red color (see left inset of Figure 3c) similar to the solid powders. Exposure of the solutions to UV light evidence an orange emission (see right inset of Figure 3c). The UV–vis electronic absorption spectra of (*S*,*S*)-**NR-9** exhibited an intense absorption with three major bands at 396, 476, and 576 nm (Figure 3c). These bands can be attributed to the central pyrene-coronene-pyrene core in agreement with the spectra of **NR-7** (Figure S1) and coronene bisimides.^64^ Meanwhile the bands corresponding to the BINAM residues overlap with those of the central core in the region between 300 and 380 nm (Figure S1). An optical HOMO–LUMO gap (*E*_gap_) of 2.1 eV was estimated from the onset of the lowest energy absorption band (Table S1). The emission spectrum of (*S*,*S*)-**NR-9** show a fluorescence band with maxima at 585 nm and with clear vibronic features that mirror the lowest energy band with a quantum yield of 27%, and a Stokes shift of 267 cm^–1^ (Figure 3c). The fluorescence energy is consistent with the orange emission observed. The redox properties of **NR-9** were investigated by cyclic voltammetry in CH_2_Cl_2_ with *n*Bu_4_PF_6_ as the supporting electrolyte (Figure 3d). (*S*,*S*)-**NR-9** exhibited three reduction waves with half-wave potentials (*E*_1/2_) at −1.31, −1.59, and a peak potential (*E*_p_) at −1.99 V versus Fc/Fc^+^, respectively. An electrochemical LUMO or electron affinity of (*S*,*S*)-**NR-9** (*E*_LUMO_) of −3.6 eV was estimated from the onset of the first reduction potential.

### Conformational Analysis

Calculations (B3LYP-6-31G(d,p)) were carried out to shine light on the conformational landscape of **NR-9**. First, we focused on a model compound of **NR-7**, namely **NR-7-H**, in which the *tert*-butyl and 1-hexylheptyl groups have been replaced respectively by hydrogen atoms and methyl groups for simplicity and computational efficiency. The calculations evidence two conformations accessible at 25 °C in constant exchange, namely one alternated (*meso*) and one helical with two enantiomers (*P* and *M*) with very similar energies (Figure 2a). The alternated conformer is the most stable and the most populated (64%), while the helical conformer is slightly less stable (+0.34 kcal/mol) with a population (36%) that is equally distributed between the two helical enantiomers.

**Figure 4:**
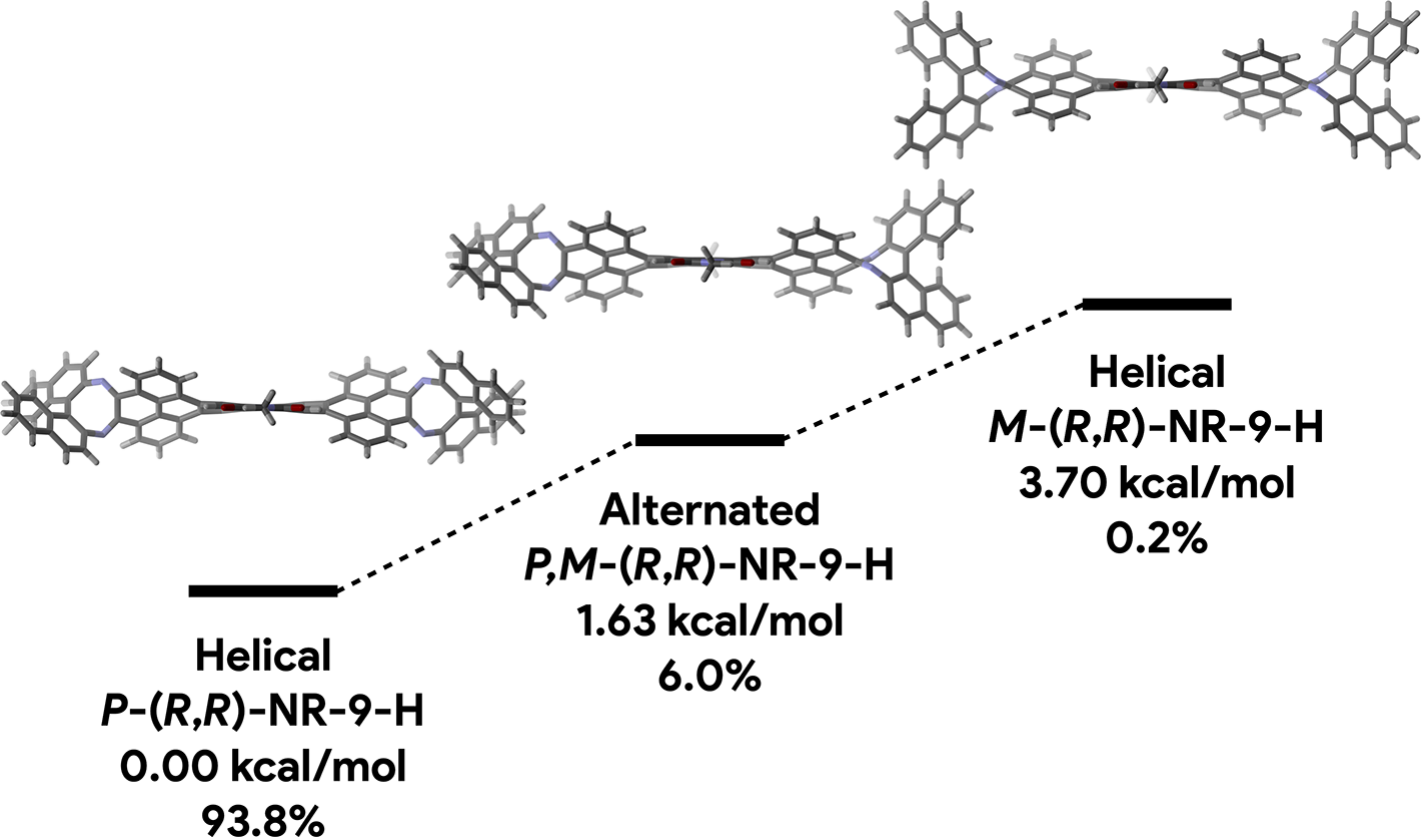
Calculated conformations, free energies, and percentual relative populations of (*R*,*R*)-**NR-9-H** at 25 °C (B3LYP-6-31G(d,p)).

A completely different trend was observed on the model **NR-9-H**, in which the *tert*-butyl and 1-hexylheptyl groups of **NR-9** have also been replaced respectively by hydrogen atoms and methyl groups. Three conformations were found to be accessible at 25 °C (Figure 4), a helical conformation with the same handedness as the chiral strain (*P*-(*R*,*R*)-**NR-9-H**), an alternated conformation (*P*,*M*-(*R*,*R*)-**NR-9-H**), and a helical conformation with the opposite handedness of that of the chiral strain (*M*-(*R*,*R*)-**NR-9-H**). The helical *P*-(*R*,*R*)-**NR-9-H** conformation is the most stable, followed by the alternated *P*,*M*-(*R*,*R*)-**NR-9-H** (+1.63 kcal/mol) and the helical *M*-(*R*,*R*)-**NR-9-H** (+3.70 kcal/mol). The relative populations evidence how the chiral ring strain generated by the diazacyclooctatetraene rings induces almost exclusively the formation of the *P*-(*R*,*R*)-**NR-9-H** conformation (93.8%), whereas the contributions of the alternated *P*,*M*-(*R*,*R*)-**NR-9-H** (6.0%) and of the *M*-(*R*,*R*)-**NR-9-H** (0.2%) conformations are almost residual. The fusion of the (*S*)-(−)-BINAM enantiomer induces helicity in the opposite direction generating again almost exclusively the *M*-(*S*,*S*)-**NR-9-H** conformer with the same degree of conformational discrimination.

### Chiroptical Properties and Absolute Configuration

To confirm experimentally the theoretical conformational analysis, the chiroptical properties of the NRs were measured by circular dichroism (CD) measurements and the CD spectra were compared with the calculated CD spectra from the simulated conformations.

The CD spectrum of **NR-7** exhibited no Cotton effect (Figure S2), which is consistent with the constant interconversion between the helical and alternated conformations observed in the calculations (Figure 2a).

**Figure 5:**
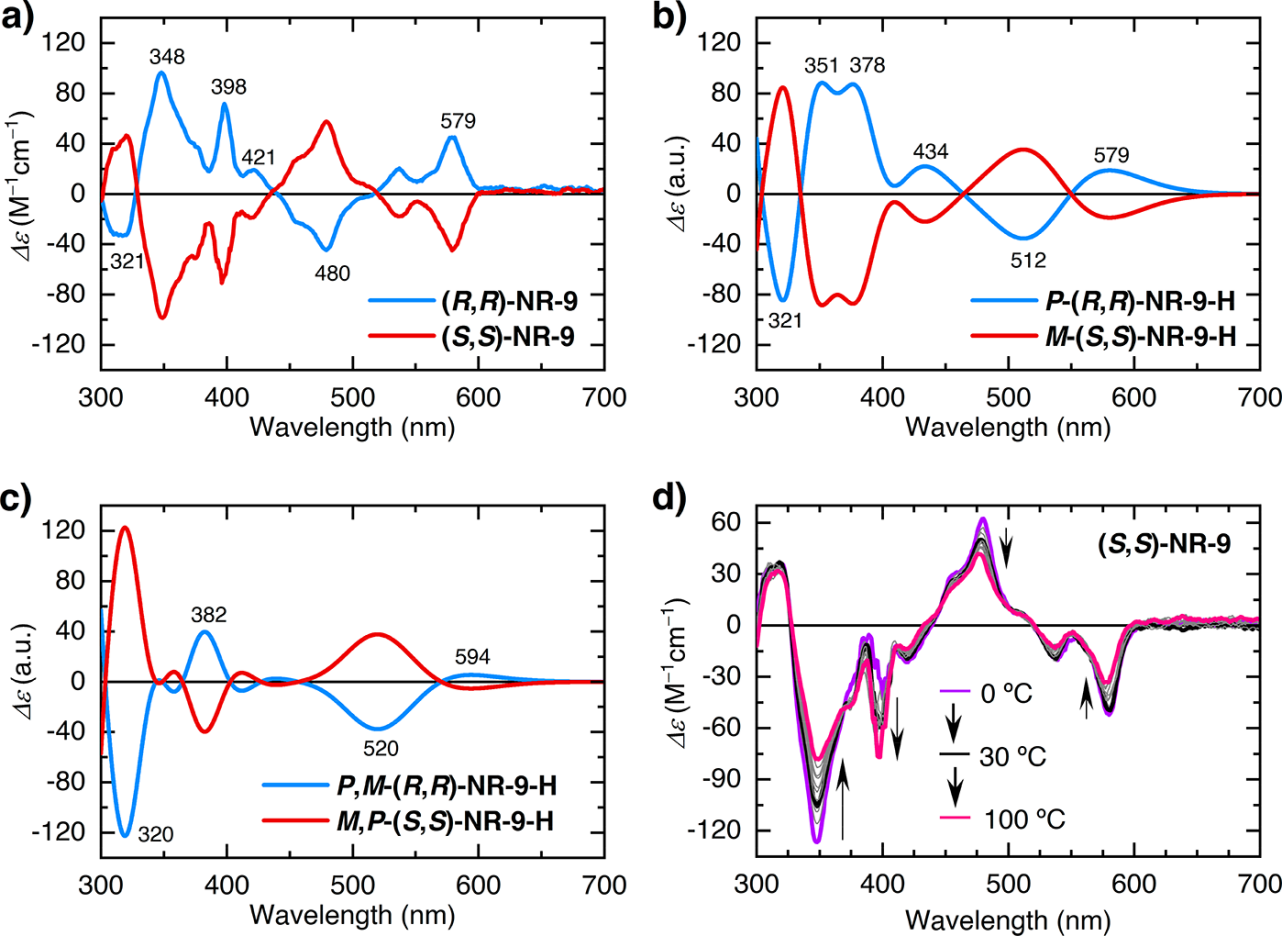
(a) CD spectra of (*R*,*R*)-**NR-9** and (*S*,*S*)-**NR-9** (10 μM, 1 cm path length) in toluene. (b) Simulated CD spectra of *P*-(*R*,*R*)-**NR-9-H** and *M*-(*S*,*S*)-**NR-9-H**. (c) Simulated CD of *P*,*M*-(*R*,*R*)-**NR-9-H** and *M*,*P*-(*S*,*S*)-**NR-9-H**. (d) VT CD spectra of (*S*,*S*)-**NR-9** in toluene.

Conversely, both (*R*,*R*)-**NR-9** and (*S*,*S*)-**NR-9** enantiomers exhibited mirror-image CD spectral patterns in a wavelength range between 300 and 600 nm (Figure 5a), in agreement with the absorption spectra, and Δε values that reach ±100 M^–1^ cm^–1^. The theoretical spectra of the homochiral helical conformers of *P*-(*R*,*R*)-**NR-9-H** and *M*-(*S*,*S*)-**NR-9-H** (Figure 5b) are in excellent agreement with the experimental ones (Figure 5a), whereas a completely different CD pattern has been obtained for the alternated conformers *P*,*M*-(*R*,*R*)-**NR-9-H** and *M*,*P*-(*S*,*S*)-**NR-9-H** (Figure 5c). Also, the simulated CD spectrum of the 94:6 *P*-(*R*,*R*)-**NR-9-H**/*P*,*M*-(*R*,*R*)-**NR-9-H** mixture predicted by the calculations at 25 °C shows a CD spectrum (Figure S3) with nearly no differences to that of the homochiral *P*-(*R*,*R*)-**NR-9-H**. This unambiguously confirms that the chiral 8-membered ring induces almost exclusively the formation of the helical conformer of the same handedness as the chiral strain.

To further verify these assignments, we synthesized and studied (*R*,*S*)-**NR-9** (Scheme S1) with two BINAM residues of opposite chirality as a reference compound. Theoretical calculations (Figure S4) evidence a major alternated *meso* conformer (*meso***-***P*,*M*-(*R*,*S*)-**NR-9-H**, 96.3%) two residual conformers, namely an asymmetric (*P*-(*R*,*S*)-**NR-9-H**, 3.4%) and a *meso* alternated (*meso***-***M*,*P*-(*R*,*S*)-**NR-9-H**, 0.2%) conformer. The CD experimental spectrum of the (*R*,*S*)-**NR-9** exhibits no Cotton effect (Figure S5), which is again in agreement with the simulated CD spectrum of the major *meso***-***P*,*M*-(*R*,*S*)-**NR-9-H** conformer that also shows no dichroic signals (Figure S5).

To study the effects of the temperature on the populations of the different conformational isomers, variable temperature CD measurements were carried out on (*S*,*S*)-**NR-9** between 0 and 100 °C (Figure 5d). This temperature window falls within the configurational stability temperature of BINAM (up to 170 °C^65^). Upon cooling, the CD spectra show the gradual increase of the intensity of the bands at 348, 480, and 579 nm, and the attenuation of the band at 398 nm, whereas upon heating, spectral changes in the opposite direction are observed. Most importantly, the intensity of the original dichroic signals is restored after bringing the sample back to 25 °C. This reversibility confirms that no racemization has taken place at the BINAM residues, and therefore, that all the CD spectral changes with respect to the temperature are the result of the dynamic nature of the molecular NRs. In addition, UV–vis electronic absorption spectra of (*S*,*S*)-**NR-9** at different temperatures (Figure S6) show virtually no differences. This confirms that the observed changes on the CD spectra at different temperatures are not related to electronic effects but to changes on the relative populations of the conformers. Theoretical calculations show that the observed changes of the CD spectra with respect to the temperature are consistent with the positive conformational discrimination in favor of the helical *M*-(*S*,*S*)-**NR-9-H** conformer at temperatures below room temperature (95.1% at 0 °C) (Table S2). Meanwhile, the discrimination toward the helical *M*-(*S*,*S*)-**NR-9-H** conformer decreases at increasing temperatures above room temperature (89.4% at 100 °C) in favor of the *M*,*P*-(*S*,*S*)-**NR-9-H** and the *P*-(*S*,*S*)-**NR-9-H** conformers (Table S2). The simulated CD spectra calculated with increasing population ratios of *M*-(*S*,*S*)-**NR-9-H** show the same trend as that observed experimentally (Figure S7). Yet, the subtle computed free energy difference, 2 kcal/mol, appears to be overestimated, which may be connected to the lack of anharmonic effects or other dynamic or solvent related effects in our model (Table S3).

### Structural Analysis and Electronic Structure

**Figure 6:**
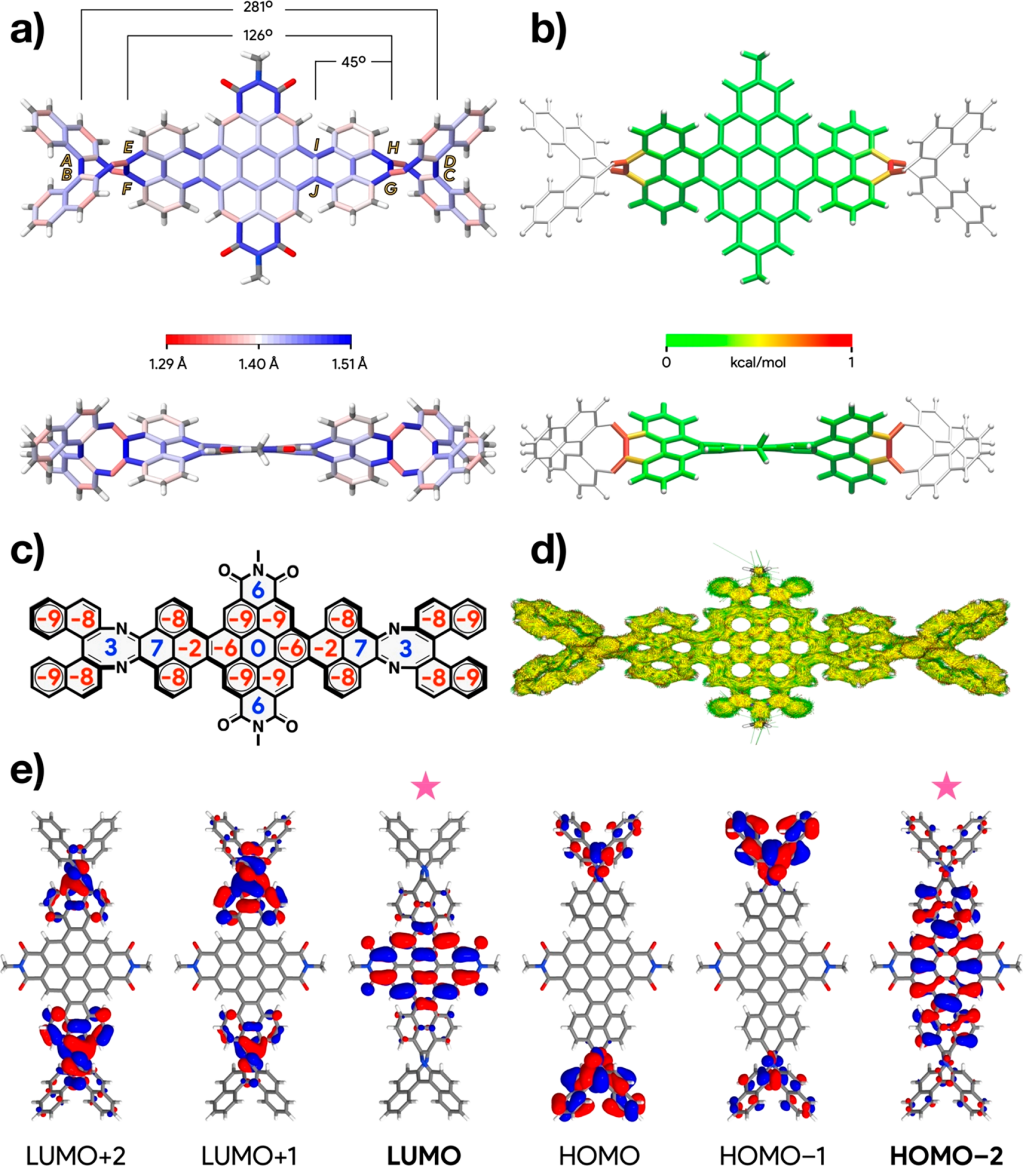
(a) Bond length plots, (b) strain plots, (c) NICS(0) values, (d) ACID plot, and (e) orbitals of *M*-(*S*,*S*)-**NR-9-H**. The orbitals highlighted with stars in (a) indicate those involved in the lowest energy transition.

The excellent correlation between experimental and calculated CD spectra allows us to get a direct insight into the structure of the most stable homochiral helical conformation of **NR-9**. The model of *M*-(*S*,*S*)-**NR-9-H** (Figure 6a,b) shows a highly twisted helical conformation with an end-to-end twist angle of 281° from the diazacyclooctatetraene ends (Ø_ABCD_, lettering shown in Figure 6a). The pyrene-to-pyrene end-to-end twist angle (Ø_EFGH_ = 126°) and the pyrene end-to-end twist angle (Ø_GHIJ_ = 45°) are higher by approximately a factor of 2 than those observed on the less-stable helical conformer of **NR-7-H** (Ø_EFGH_ = 68° and Ø_GHIJ_ = 17°). The higher twist angle values for *M*-(*S*,*S*)-**NR-9-H** are the result of the chiral strain generated by the fusion of the oversized 8-membered diazacyclooctatetraene rings and of the additional torsion generated by the homochiral BINAM residues. For instance, the bond-length plots show larger bond distances (shown in blue in Figure 6a) at the diazacyclooctatetraene-pyrene and pyrene-coronene junctions. Also, the strain plots (StrainViz^66^) show a high strain (shown in orange and red in Figure 6b) at the diazacyclooctatetraene-pyrene junctions. The propagation of such chiral strain along the NR’s backbone stabilizes the helical conformation of the same handedness as the chiral strain.

On the basis of the bond length alternation (Figure 6a) and the nucleus-independent chemical shift (NICS(0)) values (Figure 6c), the dominant resonance structure in *M*-(*S*,*S*)-**NR-9-H** is best represented by a coronene group (3 sextets) in the coronene residues, two biphenyl groups (2 sextets) in the pyrene residues, a cyclooctatetraene group (antiaromatic) in the diazacyclooctatetraene residue, and two naphthalene groups (1 sextet) in the BINAM residues. For instance, negative NICS(0) values (Figure 6c) were found on almost all the rings of the coronene pyrene and naphthalene residues (shown in red). Also, the diazacyclooctatetraene rings, linearly annulated pyrene rings, and the central coronene rings show positive and nearly positive values (shown in blue). The anisotropy of the induced current density (ACID) plots of *M*-(*S*,*S*)-**NR-9-H** (Figure 6d) are also consistent with this assignment and show a diamagnetic current that goes around the NR edges.

To shine additional light on the optoelectronic properties, DFT calculations (B3LYP-6-31G(d,p)) were carried out on *M*-(*S*,*S*)-**NR-9-H**. The computed *E*_gap_ (2.38 eV) and *E*_LUMO_ (−3.17 eV) for *M*-(*S*,*S*)-**NR-9-H** are similar to the experimental ones (Table S4). TD-DFT (Table S5) reveal that the lowest energy excitation originates from a HOMO–2 → LUMO transition, as both HOMO → LUMO and HOMO–1 → LUMO excitations are dark. The HOMO–2 is delocalized across the whole aromatic core with most of the electron density located over the pyrene-coronene-pyrene residues, and despite the highly twisted structure it shows some electron density over the diazacyclooctatetraene and binaphthyl residues (Figure 6e), whereas the LUMO is mostly localized over the coronene bisimide residue.

The similar bond lengths (Figure S8), strain plot (Figure S9), NICS(0) values (Figure S10), ACID plot (Figure S11), and orbital shapes and energies (Figure S12 and Tables S4–S5) observed in the alternated *M*,*P*-(*S*,*S*)-**NR-9-H** conformer indicate that there is not any electronic contribution to the relative conformer populations, and therefore, that chiral induction is only a strain-induced process.

## Conclusions

We have reported an unprecedented approach to induce single-handed helicity in conformationally flexible NRs with an excellent degree of conformational discrimination. Such chiral induction is the result of the chiral strain generated by fusing two oversized chiral rings and of the propagation of that chiral strain along the NR’s backbone. The chiral NRs produce dichroic signals in a broad spectral range up to 600 nm. The simulation of the experimental dichroic spectral patterns of the chiral NRs allow confirmation that the chiral 8-membered rings stabilize the helical conformation of the same handedness as the chiral strain. Overall, this asymmetric approach paves the way for the synthesis of more complex homochiral nanographenes, which in turn will enable further developments in electronic and spintronic applications that exploit the absorption and emission of circularly polarized light and chiral induced spin selectivity.

## Supporting Information Available

Experimental details of the synthesis, characterization, and calculations (PDF)

## Abbreviations

NR: graphene nanoribbon
HPLC: high-performance liquid chromatography
BINAM: 1,1′-binaphthyl-2,2′-diamine
THF: tetrahydrofuran
DMF: *N*,*N*-dimethylformamide
NMP: *N*-methyl pyrrolidone
NMR: nuclear magnetic resonance
HR MALDI-TOF-MS: high-resolution matrix-assisted laser desorption/ionization time-of-flight mass spectrometry
CD: circular dichroism
VT-CD: variable temperature circular dichroism
NICS: nuclear independent chemical shifts
ACID: anisotropy of the induced current density
DFT: density functional theory
HOMO: highest occupied molecular orbital
LUMO: lowest unoccupied molecular orbital.
