# Supplementary material for: Inducing Single-Handed Helicity in a Twisted Molecular Nanoribbon
Source: J Am Chem Soc. 2022 Jan 31;144(6):2765–74. doi: 10.1021/jacs.1c12385 (PMC8855342; doi:10.1021/jacs.1c12385)
Supplement: Supplementary file 1 — ja1c12385_si_001.pdf [file ja1c12385_si_001.pdf]

Inducing Single-Handed Helicity in a Twisted Molecular Nanoribbon

Rajeev K. Dubey,[†] Manuel Melle-Franco,[≠]* Aurelio Mateo-Alonso[†#]*

[†]*POLYMAT, University of the Basque Country UPV/EHU, Avenida Tolosa 72, 20018 Donostia-San Sebastian (Spain)*

[≠]*CICECO, Aveiro Institute of Materials, Department of Chemistry, University of Aveiro, 3810-193 Aveiro (Portugal)*

[#]*Ikerbasque, Basque Foundation for Science, 48009 Bilbao (Spain)*

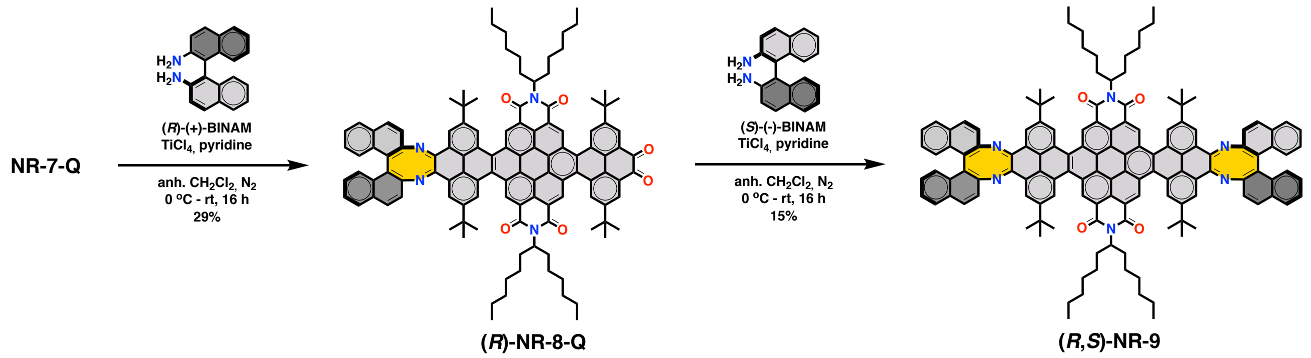

Scheme S1. Synthesis of ***(R,S)*-NR-9**.

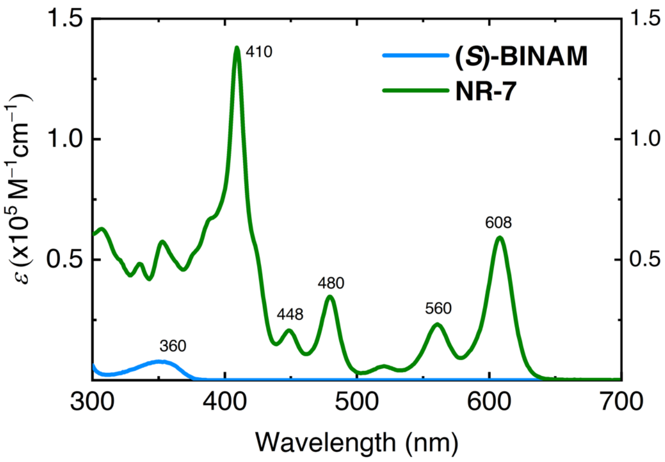

Figure S1. UV-vis spectra of **(*S*)-BINAM** and **NR-7** in toluene.

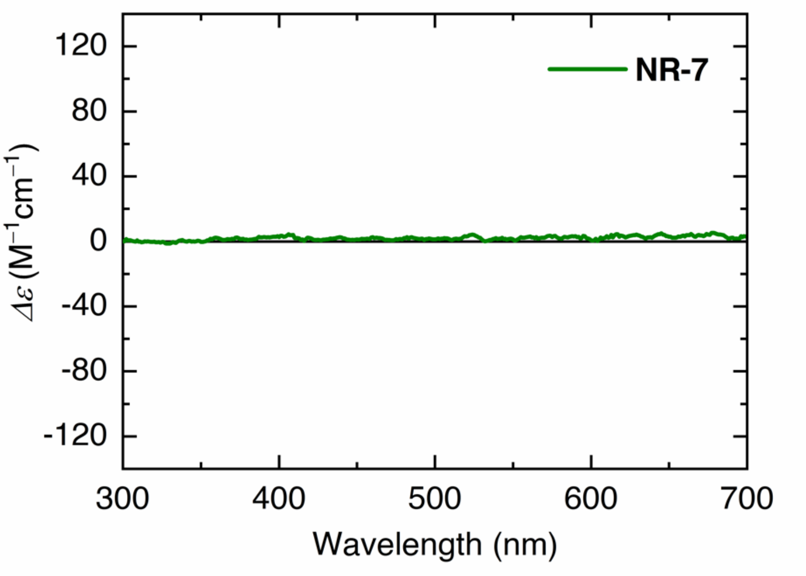

Figure S2. CD of **NR-7** in toluene (10 µM, 1 cm path length).

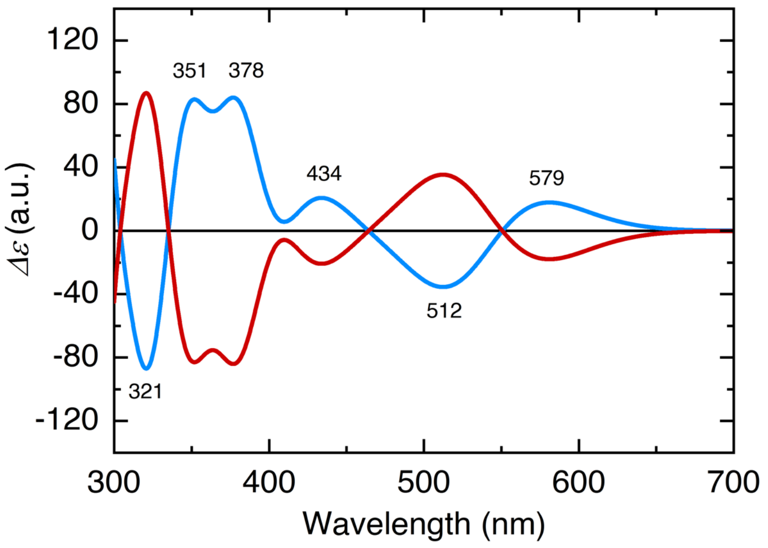

Figure S3. Simulated CD spectra of two 94:6 mixtures of ***P*-(*R*,*R*)-NR-9-H** / ***P*,*M*-(*R*,*R*)-NR-9-H** (blue trace) and ***M*-(*S*,*S*)-NR-9-H** / ***M*,*P*-(*S*,*S*)-NR-9-H** (red trace).

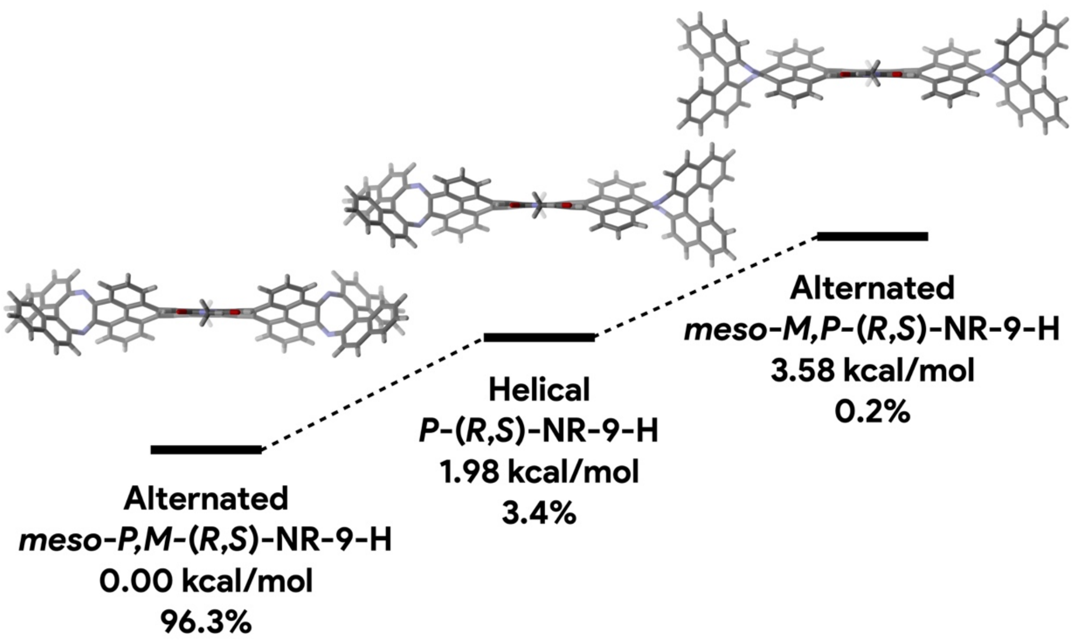

Figure S4. Calculated conformations, free energies and percentual relative populations of ***(R,S)*-NR-9-H** at 25 ºC (B3LYP-6-31G(d,p)).

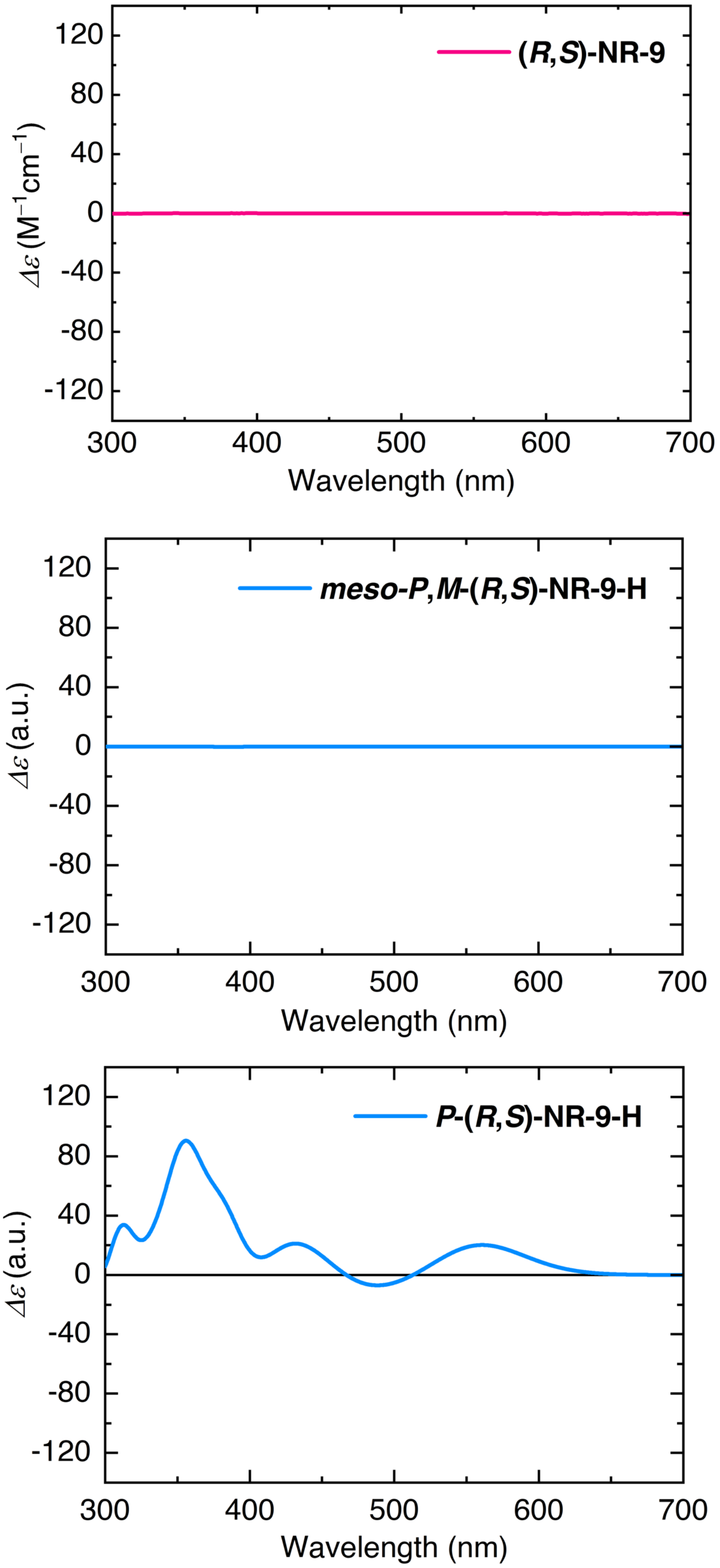

Figure S5. (top) CD spectrum of **(*R*,*S*)-NR-9** in toluene (11 μM, 1 cm path length). Simulated CD spectrum of (center) ***meso-P*,*M*-(*R*,*S*)-NR-9-H** and of (bottom) ***P*-(*R*,*S*)-NR-9-H**.

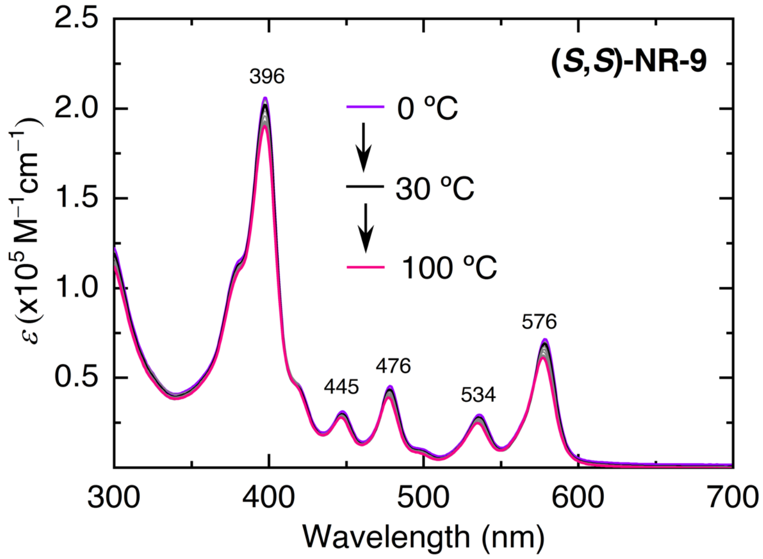

Figure S6. Variable temperature UV-vis electronic absorption spectra of **(*S*,*S*)-NR-9** in toluene (8.0 μM, 1 cm path length).

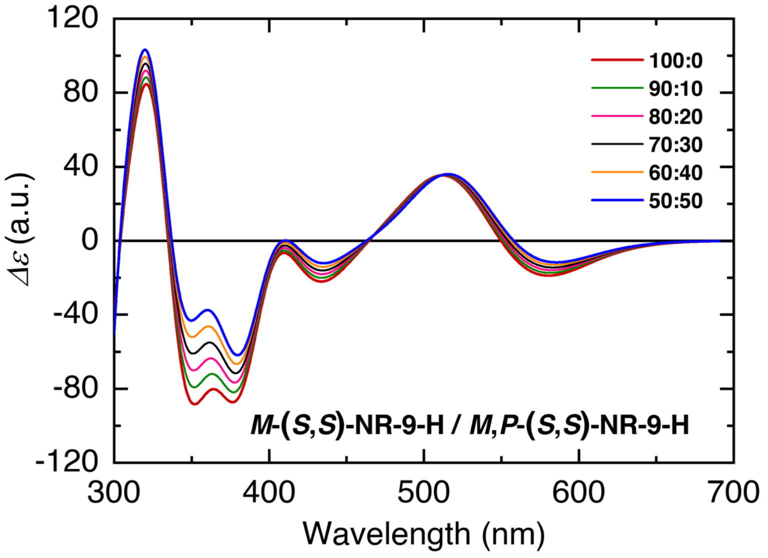

Figure S7. CD spectrum with different ratios of ***M*-(*S*,*S*)-NR-9-H** and ***M*,*P*-(*S*,*S*)-NR-9-H**.

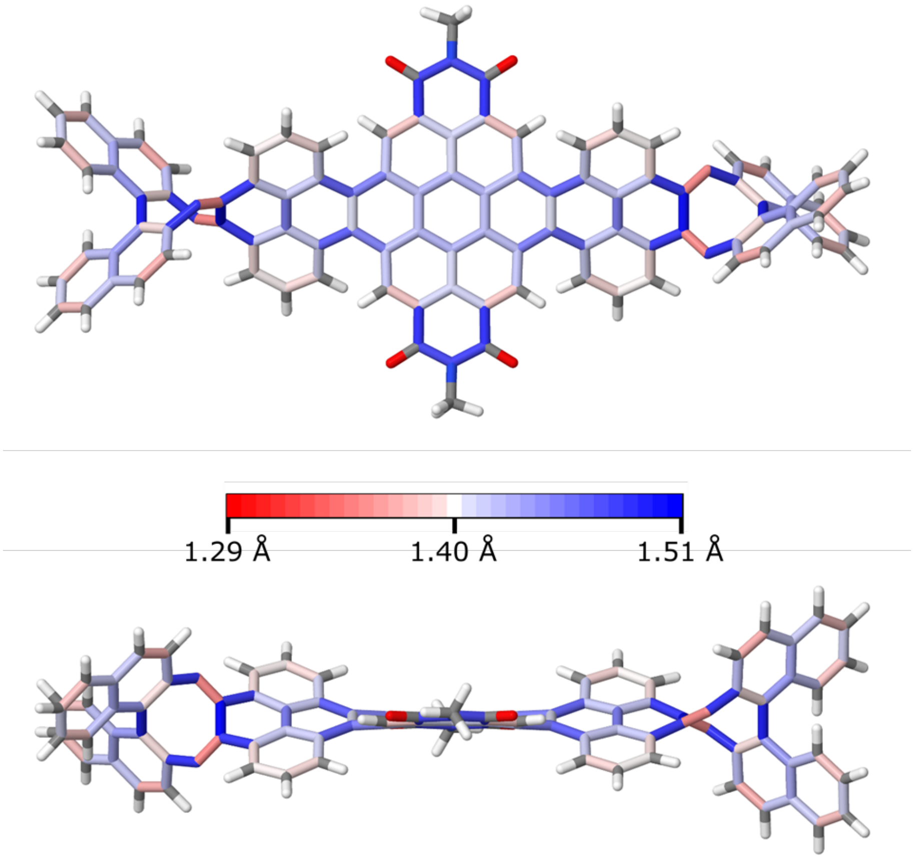

Figure S8. Bond length plots of ***M*,*P*-(*S*,*S*)-NR-9-H**.

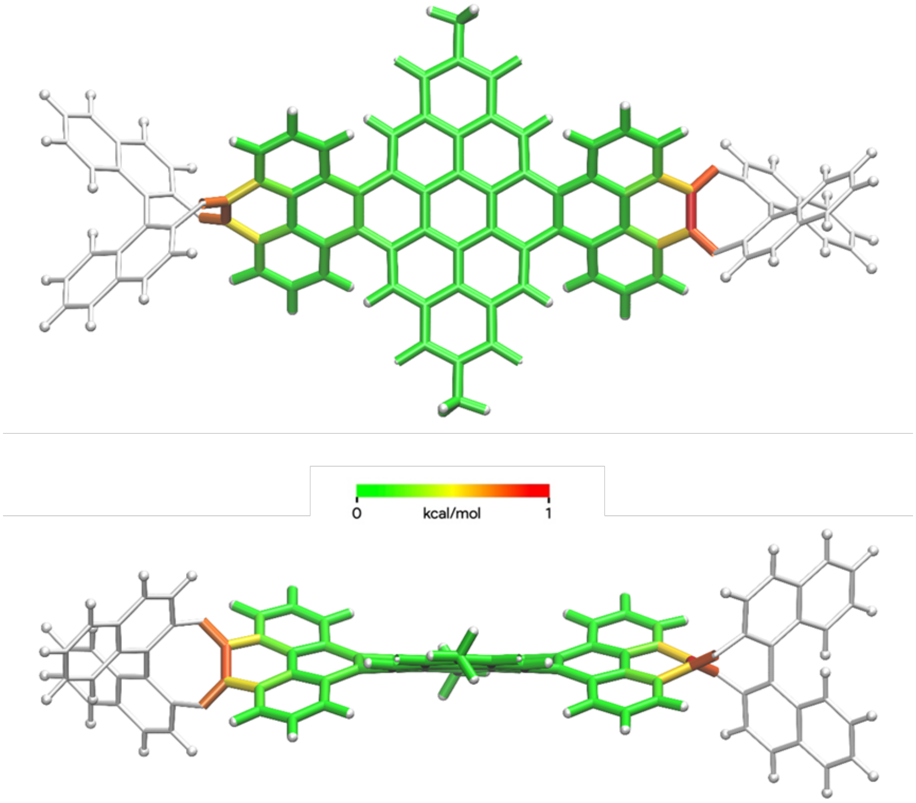

Figure S9. Strain plots of ***M*,*P*-(*S*,*S*)-NR-9-H**.

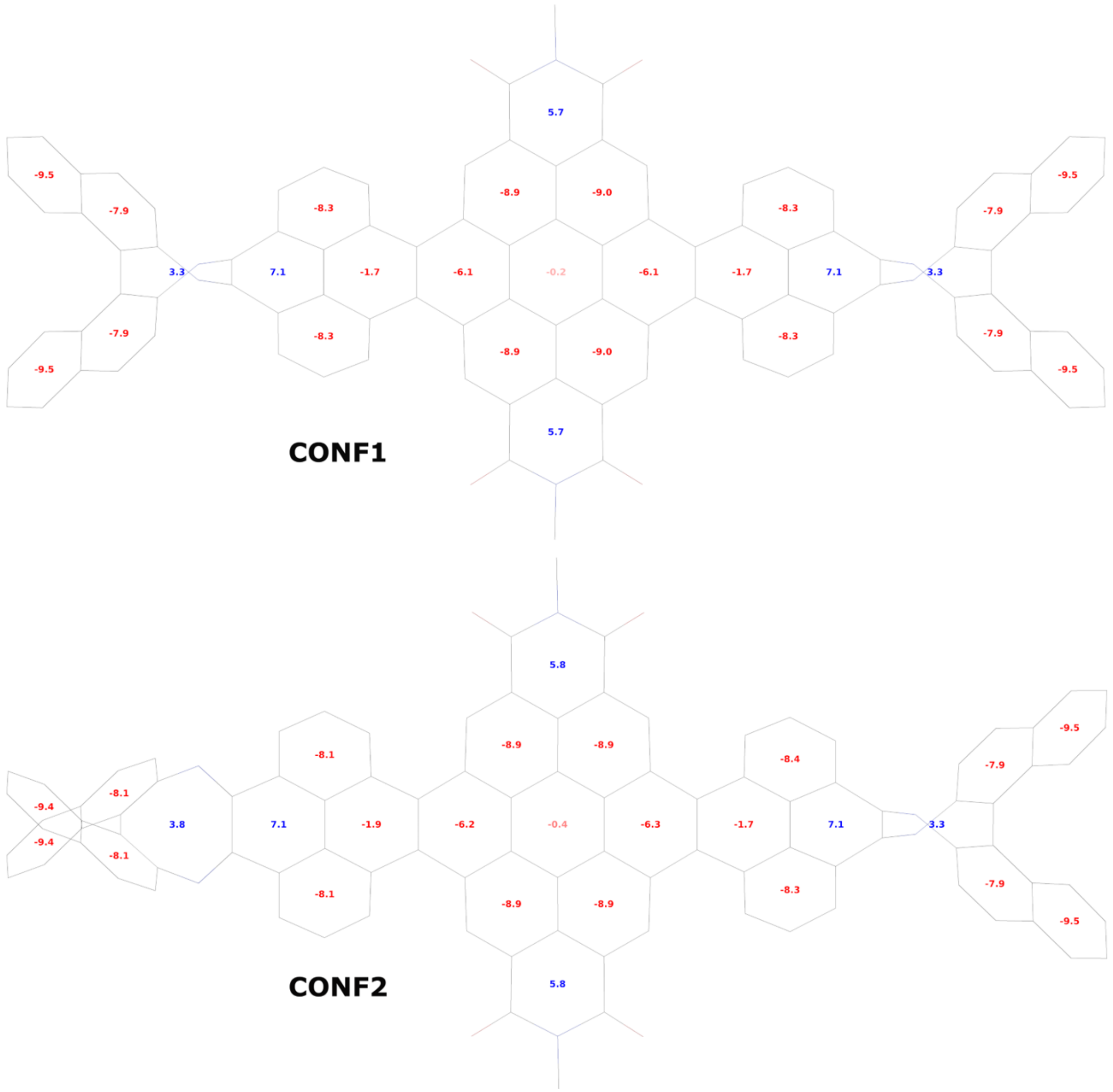

Figure S10. NICS(0) values of ***M*-(*S*,*S*)-NR-9-H** (top) and ***M*,*P*-(*S*,*S*)-NR-9-H** (bottom).

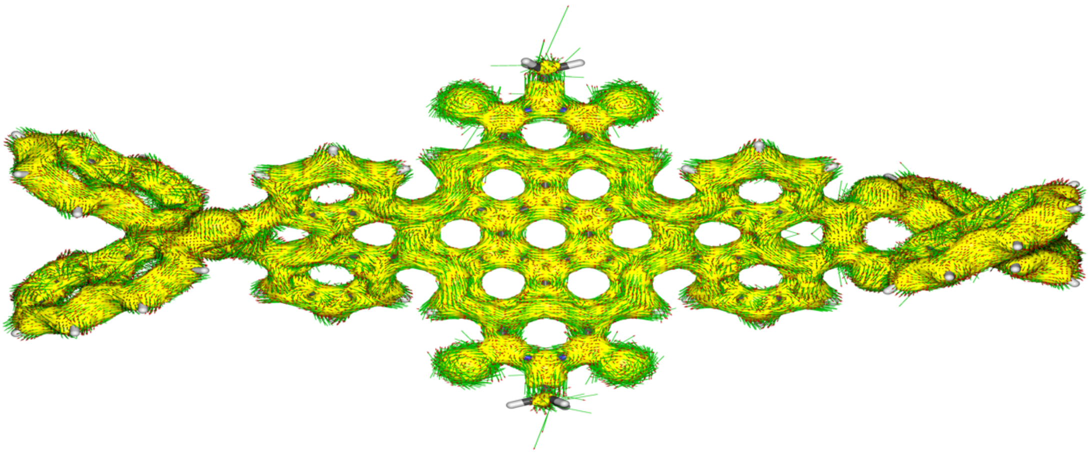

Figure S11. ACID plot of ***M*,*P*-(*S*,*S*)-NR-9-H**.

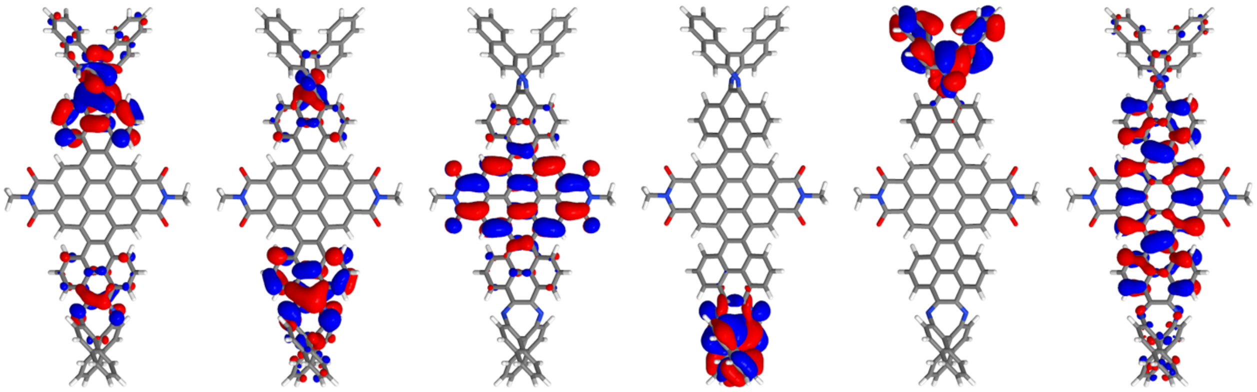

Figure S12. B3LYP-6-31G(d,p) frontier orbitals for ***M*****,*****P*****-(*****S*****,*****S*****)-NR-9-H**. From left to right: LUMO+2, LUMO+1, LUMO, HOMO, HOMO–1, HOMO–2.

Table S1. Optical and Electronic Properties of **(*S*,*S*)-NR-9**.[a,b]

Compound	λ_{abs} (ε, $M^{-1}cm^{-1}$)	λ_{em} (nm)	Φ_f[c]	E_{1red}	E_{2red}	E_{3red}	$E_{g\,(opt)}$ (eV)[d]	E_{LUMO} (eV)[e]	E_{HOMO} (eV)[f]
**(*S*,*S*)-NR-9**	396 (196700),<br>445 (30200),<br>476 (42700),<br>534 (26400),<br>576 (63700)	585	0.27	–1.31	–1.59	–1.99	2.09	–3.60	–5.69

[a]The absorption and emission were measured in toluene. [b]The redox potentials (V vs Fc/Fc^+) obtained by cyclic voltammetry in dichloromethane (Scan rate = 50 mV/s). The potentials are reported as $E_{1/2} = (E_p^a + E_p^c)/2$ and quoted to the nearest 0.01 V. [c]Fluorescence quantum yield. [d]Optical band gap calculated using equation $E_g = hc/\lambda a.e. \approx 1240/\lambda ae$ (nm); Where λae denotes the absorption edge wavelength in nm, obtained from offset wavelength derived from the lowest energy absorption band in DCM. [e]The LUMO levels were estimated from the onset of the first reduction potential $E_{LUMO} = -(E_{1red(ONSET)} + 4.8eV)$. [f]Estimated from $E_{HOMO} = E_{LUMO} - E_g$.

Table S2. Relative populations of **NR-9-H** calculated at different temperatures.

**Conformer**	**Electronic Energy (kcal/mol)**	**Free Energy (kcal/mol)**	**Relative populations**		
			**0 °C**	**25 °C**	**100 °C**
***P*-(*R*,*R*)-NR-9-H (*M*-(*S*,*S*)-NR-9-H)**	0.00	0.00	**95.1%**	**93.8%**	**89.4%**
***P*,*M*-(*R*,*R*)-NR-9-H (*M*,*P*-(*S*,*S*)-NR-9-H)**	1.70	1.63	4.8%	6.0%	10.0%
***M*-(*R*,*R*)-NR-9-H (*P*-(*S*,*S*)-NR-9-H)**	3.76	3.70	0.1%	0.2%	0.6%

Table S3. Relative electronic energies of **NR-9-H** calculated with different methods (kcal/mol).

**Conformer**	**B3LYP-6-31G(d,p)**	**B3LYP-6-311G+(d,p) /B3LYP-6-31G(d,p)**	**DLPNO-CSSD(T)-def2-SVP /B3LYP-6-31G(d,p)**	**M06-2x-6-31G(d,p)**
***P*-(*R*,*R*)-NR-9-H (*M*-(*S*,*S*)-NR-9-H)**	0.00	0.00	0.00	0.00
***P*,*M*-(*R*,*R*)-NR-9-H (*M*,*P*-(*S*,*S*)-NR-9-H)**	1.70	1.83	1.68	2.16
***M*-(*R*,*R*)-NR-9-H (*P*-(*S*,*S*)-NR-9-H)**	3.76	4.09	4.36	4.49

Table S4. Orbital energies for the three possible conformers of **NR-9-H** (eV).

**B3LYP-6-31G(d,p) Vacuum**																		
**Conformer**									**LUMO**	**HOMO**	**H-1**							**Gap**
***P*-(*R*,*R*)-NR-9-H (*M*-(*S*,*S*)-NR-9-H)**	-1.21	-1.46	-1.66	-1.76	-1.86	-2.37	-2.42	-2.42	-3.17	-5.55	-5.55	-5.77	-5.86	-5.88	-6.08	-6.19	-6.21	2.38
***P*,*M*-(*R*,*R*)-NR-9-H (*M*,*P*-(*S*,*S*)-NR-9-H)**	-1.23	-1.48	-1.68	-1.76	-1.87	-2.38	-2.42	-2.42	-3.16	-5.54	-5.55	-5.76	-5.86	-5.88	-6.07	-6.19	-6.21	2.38
***M*-(*R*,*R*)-NR-9-H (*P*-(*S*,*S*)-NR-9-H)**	-1.26	-1.49	-1.66	-1.77	-1.88	-2.37	-2.42	-2.42	-3.16	-5.54	-5.54	-5.75	-5.86	-5.88	-6.06	-6.18	-6.20	2.38

Table S5. TD-DFT first active excitation for the three possible conformers of **NR-9-H**.

**B3LYP-6-31G(d,p)**			
**Conformer**	**First Transition (eV)**	**Osc. Strength**	**Contributions**
***P*-(*R*,*R*)-NR-9-H (*M*-(*S*,*S*)-NR-9-H)**	2.21	0.52	H-2->LUMO (91%), H-5->L+3 (4%), H-4->LUMO (4%)
***P*,*M*-(*R*,*R*)-NR-9-H (*M*,*P*-(*S*,*S*)-NR-9-H)**	2.22	0.54	H-2->LUMO (91%), H-5->L+3 (4%), H-4->LUMO (3%)
***M*-(*R*,*R*)-NR-9-H (*P*-(*S*,*S*)-NR-9-H)**	2.19	0.54	H-2->LUMO (92%), H-5->L+3 (4%), H-4->LUMO (3%)

Materials and methods

Reagents. All the commercial reagents utilized in the synthesis were used as received. The compounds NR-7 and NR-7-Q were synthesized following a reported procedure.[1]

Synthesis. All the reactions were performed in an oven-dried round-bottom flask or a Schlenk tube.

Purification. The purification of the products was performed by silica-gel column chromatography under ambient conditions. The sorbent for the column chromatography (silica gel 60, 0.04–0.06 mm, 230−400 mesh) and the TLC plates were purchased from commercial suppliers. The monitoring of the purification process was performed by thin-layer chromatography on TLC plates.

Characterization. The NMR spectra were recorded with 400 or 500 MHz pulsed Fourier transform NMR spectrometer in deuterated solvents at room temperature unless otherwise noted. The chemical shift values are given in ppm and *J* values in Hz. The mass spectra were recorded on aBruker Daltonics-Autoflex MALDI-TOF mass spectrometer by Dr. Estíbaliz González de San Román Martín. High-resolution mass spectra were recorded by Dr. Javier Calvo in reflector acquisition operation mode on UltrafleXtreme III MALDI tandem mass spectrometer (Bruker).

Cyclic voltammetry. The cyclic voltammetry was performed in a three-electrode single-compartment cell consisting of a glassy carbon working electrode, silver wire as the reference electrode, and a platinum wire as the counter electrode. The cell was connected to the computer controlled potentiostat (Princeton Applied Research - PARSTAT 2273). The measurements were carried out under N_2 atmosphere in anhydrous CH_2Cl_2 using tetrabutylammonium hexafluorophosphate (0.1 M) as the supporting electrolyte. The concentration of the prepared samples was ca. 0.5 mM. The potentials of all the reversible peaks are reported as $E_{1/2} = (E_p^a + E_p^c)/2$ in V vs Fc/Fc^+ and quoted to the nearest 0.01 V. The measurements were carried out at a scan rate of 50 mV/s. Under these experimental conditions, the ferrocene oxidation was observed at ca. 0.41 V.

Absorption and emission spectroscopy. The absorption spectra were recorded with a double beam UV/VIS/NIR spectrophotometer (PerkinElmer – Lambda 950) and the emission spectra were collected on a fluorescence spectrometer (PerkinElmer – LS 55). Fluorescence quantum yields were determined by the comparative method using cresyl violet perchlorate[2] ($\Phi_f = 0.54$ in methanol) as a standard.

Circular dichroism. The CD spectra were recorded on spectropolarimeter (J-815 Jasco) in a wavelength range of 300–800 nm with a scan speed of 500 nm/min. The sample solutions (10 μM) were prepared in spectroscopy grade toluene.

Computer models. Conformations were obtained by a conformer exploration procedure based on metadynamics, the Conformer–Rotamer Ensemble Sampling Tool (CREST),[3] which uses a Semiempirical Quantum Mechanics (SQM) Hamiltonians.[4] The obtained conformations were then refined with DFT at the B3LYP-6-31G(d,p) and, for comparison, the M06-2X-6-31g(d,p) level. The relative energies were also computed at the B3LYP-6-311+G(d,p)/B3LYP-6-31G(d,p) level and with the DLPNO-CCSD(T) Hamiltonian with the def2-SVP basis set (Table S3).[5] DFT calculations were done by Gaussian09[6], while the DLPNO-CCSD(T) were computed with Orca V5.0.1.[7] The Jmol software was used to produce figures and to graphically analyze the results.[8]

Synthetic procedures

Synthesis of (*S,S*)-NR-9:

An oven dried Schlenk tube was charged with NR-7-Q[1] (130 mg, 0.09 mmol, 1 eq.), (*S*)-(–)-1,1′-binaphthyl-2,2′-diamine (154 mg, 0.54 mmol, 6 eq.), anhydrous pyridine (142 µL, 1.76 mmol, 20 eq.), and anhydrous DCM (15 mL). The tube was sealed using a rubber septum and the resultant solution was deoxygenated by bubbling with N_2 for five minutes. Afterwards, the solution was cooled to 0 °C in an ice bath. Titanium tetrachloride (60 µL, 0.54 mmol, 6 eq.) was added and the reaction mixture was stirred for 16 h at room temperature. Then, additional DCM (40 mL) was added and the solution was washed with water (2 x 100 mL). The organic phase was collected and concentrated. The resultant solid residue was purified on silica-gel column (1:1 DCM-hexane) to afford the desired product (89 mg, 51%) as dark red crystalline solid. ^{1}H NMR (400 MHz, CD_2Cl_2): δ = 10.96 (br s, 2H), 10.93 (br s, 2H), 9.34 (s, 4H), 8.33 (s, 4H), 8.11 (d, J = 8.0 Hz, 4H), 8.07 (d, J = 8.0 Hz, 4H), 7.59 (t, J = 8.0 Hz, 4H), 7.50 (d, J = 8.0 Hz, 4H), 7.40 (t, J = 8.0 Hz, 4H), 7.28 (d, J = 8.0 Hz, 4H), 5.60–5.51 (m, 2H), 2.66–2.48 (m, 4H), 2.11–2.01 (m, 4H), 1.60 (s, 36H), 1.52–1.26 (m, 32H), 0.93–0.81 ppm (m, 12H). ^{13}C{^{1}H} NMR (126 MHz, CD_2Cl_2): δ = 166.45, 165.98, 164.80, 151.75, 150.41, 133.54, 133.09, 131.88, 131.30, 130.52, 130.35, 129.20, 128.39, 128.36, 128.24, 126.89, 126.79, 126.60, 125.34, 124.13, 123.43, 122.97, 122.71, 121.90, 120.48, 54.87, 35.61, 32.78, 32.28, 31.91, 31.81, 31.14, 29.38, 29.25, 27.01, 26.91, 22.63, 22.59, 13.85, 13.80 ppm. HRMS (MALDI-TOF): [M]$^+$ Calculated for $C_{138}H_{126}N_6O_4$, 1930.9839; found, 1930.9758.

Synthesis of (*R,R*)-NR-9:

(*R,R*)-NR-9 was achieved (95 mg, 54%) as dark red solid by using (*R*)-(+)-1,1′-binaphthyl-2,2′-diamine in place of (S)-(–)-1,1′-binaphthyl-2,2′-diamine. Otherwise, following the same procedure as described above for the synthesis of (*S,M,S*)-NR-9. ^{1}H NMR (400 MHz, CD_2Cl_2): δ = 10.96 (br s, 2H), 10.93 (br s, 2H), 9.34 (s, 4H), 8.33 (s, 4H), 8.11 (d, J = 8.0 Hz, 4H), 8.07 (d, J = 8.0 Hz, 4H), 7.59 (t, J = 8.0 Hz, 4H), 7.50 (d, J = 8.0 Hz, 4H), 7.40 (t, J = 8.0 Hz, 4H), 7.28 (d, J = 8.0 Hz, 4H), 5.60–5.51 (m, 2H), 2.66–2.48 (m, 4H), 2.11–2.01 (m, 4H), 1.60 (s, 36H), 1.52–1.26 (m, 32H), 0.93–0.81 ppm (m, 12H). ^{13}C{^{1}H} NMR (101 MHz, CD_2Cl_2): δ = 166.82, 166.24, 165.18, 152.11, 150.77, 133.90, 133.45, 132.24, 130.71, 129.57, 128.76, 128.73, 128.60, 127.25, 127.15, 126.96, 125.70, 124.49, 123.79, 123.33, 123.07, 122.27, 120.84, 55.23, 35.98, 33.14, 32.63, 32.28, 32.18, 31.51, 29.75, 29.61, 27.38, 27.27, 23.00, 22.96, 14.23, 14.18 ppm. HRMS (MALDI-TOF): [M]$^+$ Calculated for $C_{138}H_{126}N_6O_4$, 1930.9839; found, 1930.9858.

Synthesis of (*R*)-NR-8-Q:

An oven dried round-bottom flask (25 mL) was charged with NR-7-Q (130 mg, 0.09 mmol, 1 eq.), (R)-(+)-1,1′-binaphthyl-2,2′-diamine (102 mg, 0.36 mmol, 4 eq.), anhydrous pyridine (87 μL, 1.08 mmol, 12 eq.), and anhydrous DCM (15 mL). The flask was sealed using a rubber septum and the resultant solution was deoxygenated by bubbling with N_2 for a few minutes. Afterwards, the solution was cooled to 0 °C in an ice bath. Titanium tetrachloride (40 μL, 0.36 mmol, 4 eq.) was added and the reaction mixture was stirred for 16 h at room temperature. Then, additional DCM (40 mL) was added and the solution was washed with 2M HCl (2 x 100 mL) and water (1 x 100 mL). The organic phase was collected and concentrated. The resultant solid residue was purified on silica-gel column (2:1 DCM-hexane) to afford the desired product (44 mg, 29%) as dark red solid. Side-product (*R*,*R*)-NR-9 (30 mg, 17%) and unreacted NR-7-Q (40 mg, 31%) were also collected from the column. ^{1}H NMR (500 MHz, $CDCl_3$): δ = 10.99 (br s, 1H), 10.95 (br s, 1H), 10.86 (br s, 1H), 10.82 (br s, 1H), 9.48 (s, 2H), 9.27 (s, 2H), 8.87 (s, 2H), 8.32 (s, 2H), 8.07 (d, J = 10.0 Hz, 2H), 8.04 (d, J = 10.0 Hz, 2H), 7.56 (t, J = 10.0 Hz, 2H), 7.50 (d, J = 10.0 Hz, 2H), 7.39 (t, J = 10.0 Hz, 2H), 7.31 (d, J = 10.0 Hz, 2H), 5.60–5.51 (m, 2H), 2.66–2.43 (m, 4H), 2.09–2.01 (m, 4H), 1.69 (s, 18H), 1.60 (s, 18H), 1.52–1.26 (m, 32H), 0.94–0.77 ppm (m, 12H). ^{13}C{^{1}H} NMR (126 MHz, $CDCl_3$): δ = 180.96, 166.50, 166.09, 164.83, 152.31, 152.18, 150.57, 135.03, 133.78, 133.34, 132.00, 130.38, 130.20, 129.40, 129.06, 128.62, 128.37, 128.10, 128.00, 127.56, 127.18, 127.09, 126.80, 126.73, 125.51, 124.56, 124.16, 123.81, 123.41, 123.36, 122.41, 120.83, 55.30, 35.99, 35.94, 32.93, 32.50, 32.05, 31.67, 31.45, 29.56, 29.51, 27.21, 27.17, 22.85, 14.32, 14.29 ppm. HRMS (MALDI-TOF): $[M]^+$ Calculated for $C_{118}H_{114}N_4O_6$, 1683.877; found, 1683.884.

Synthesis of (*R,S*)-NR-9:

An oven dried round-bottom flask (25 mL) was charged with (*R*)-NR-8-Q (45 mg, 0.03 mmol, 1 eq.), (S)-(–)-1,1′-binaphthyl-2,2′-diamine (76 mg, 0.28 mmol, 10 eq.), anhydrous pyridine (26 μL, 0.32 mmol, 12 eq.), and anhydrous DCM (7.5 mL). The flask was sealed using a rubber septum and the resultant solution was deoxygenated by bubbling with N_2 for a two minutes. Afterwards, the solution was cooled to 0 °C in an ice bath. Titanium tetrachloride (35 μL, 0.32 mmol, 12 eq.) was added and the reaction mixture was stirred for 16 h at room temperature. Then, additional DCM (40 mL) was added and the solution was washed with 2M HCl (2 x 100 mL) and water (1 x 100 mL). The organic phase was collected and concentrated. The resultant solid residue was purified on silica-gel column (1:1 DCM-hexane) to afford the desired product (9.1 mg, 15%) as dark red solid. ^{1}H NMR (500 MHz, CD_2Cl_2): δ = 10.95 (br s, 2H), 10.91 (br s, 2H), 9.32 (s, 4H), 8.32 (s, 4H), 8.10 (d, J = 10.0 Hz, 4H), 8.06 (d, J = 10.0 Hz, 4H), 7.58 (t, J = 10.0 Hz, 4H), 7.49 (d, J = 10.0 Hz, 4H), 7.39 (t, J = 10.0 Hz, 4H), 7.27 (d, J = 10.0 Hz, 4H), 5.59–5.51 (m, 2H), 2.67–2.46 (m, 4H), 2.10–1.97 (m, 4H), 1.58 (s, 36H), 1.49–1.26 (m, 32H), 0.89–0.81 ppm (m, 12H). ^{13}C{^{1}H} NMR (126 MHz, CD_2Cl_2): δ = 166.45, 166.01, 164.85, 151.74, 150.40, 133.54, 133.09, 131.88, 131.28, 130.55, 130.33, 129.19, 128.39,

128.36, 128.23, 126.89, 126.86, 126.78, 126.59, 125.33, 124.12, 123.42, 122.96, 122.70, 121.90, 120.48, 54.87, 35.61, 32.77, 32.27, 31.91, 31.80, 31.13, 29.37, 29.24, 27.00, 26.90, 22.62, 22.59, 13.85, 13.80 ppm. HRMS (MALDI-TOF): $[M]^+$ Calculated for $C_{138}H_{126}N_6O_4$, 1931.986; found, 1931.980.

NMR spectra

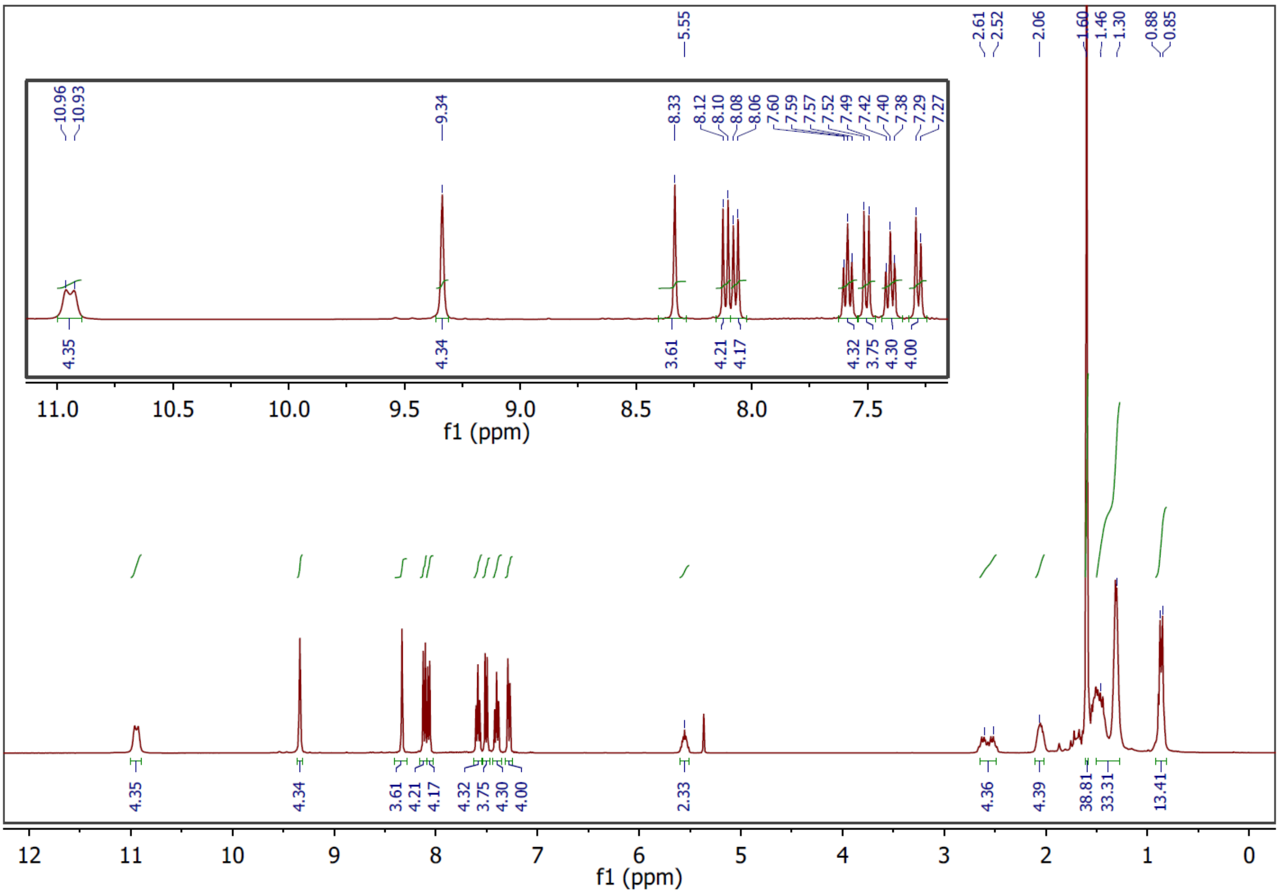

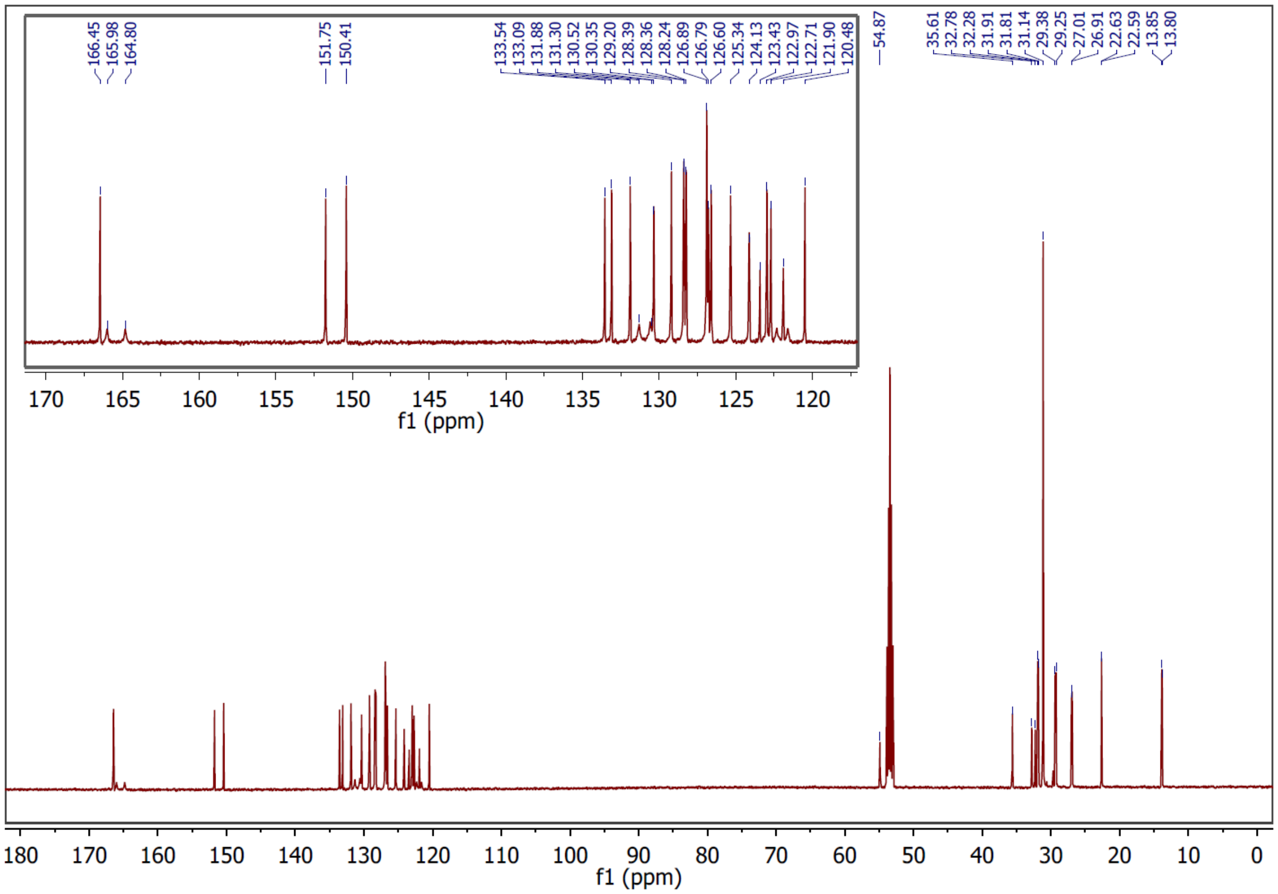

Figure S13. ^{1}H and ^{13}C NMR spectra of (***S*,*S***)**-NR-9**.

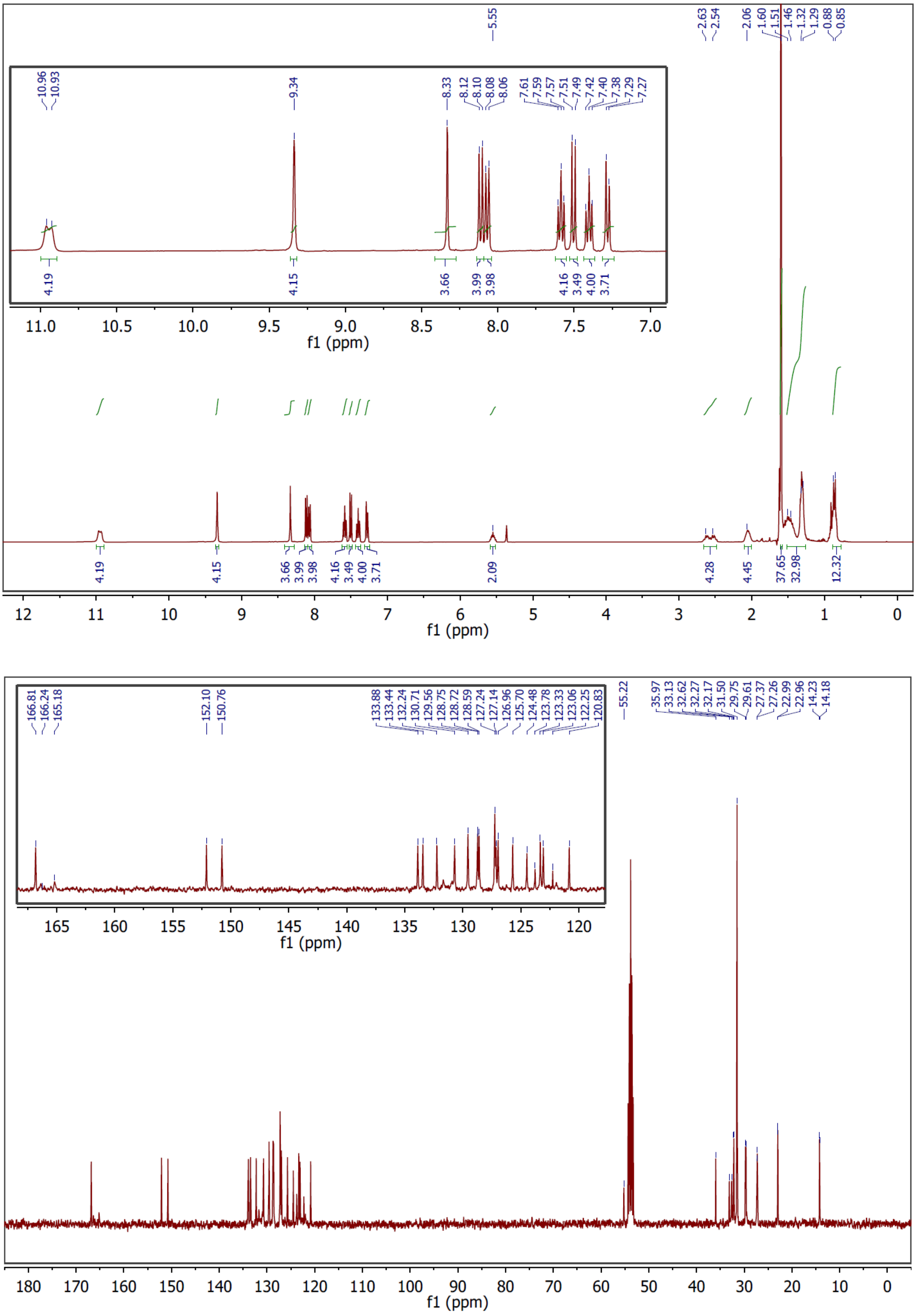

Figure S14. ^{1}H and ^{13}C NMR spectra of (***R*,*R***)-**NR-9**.

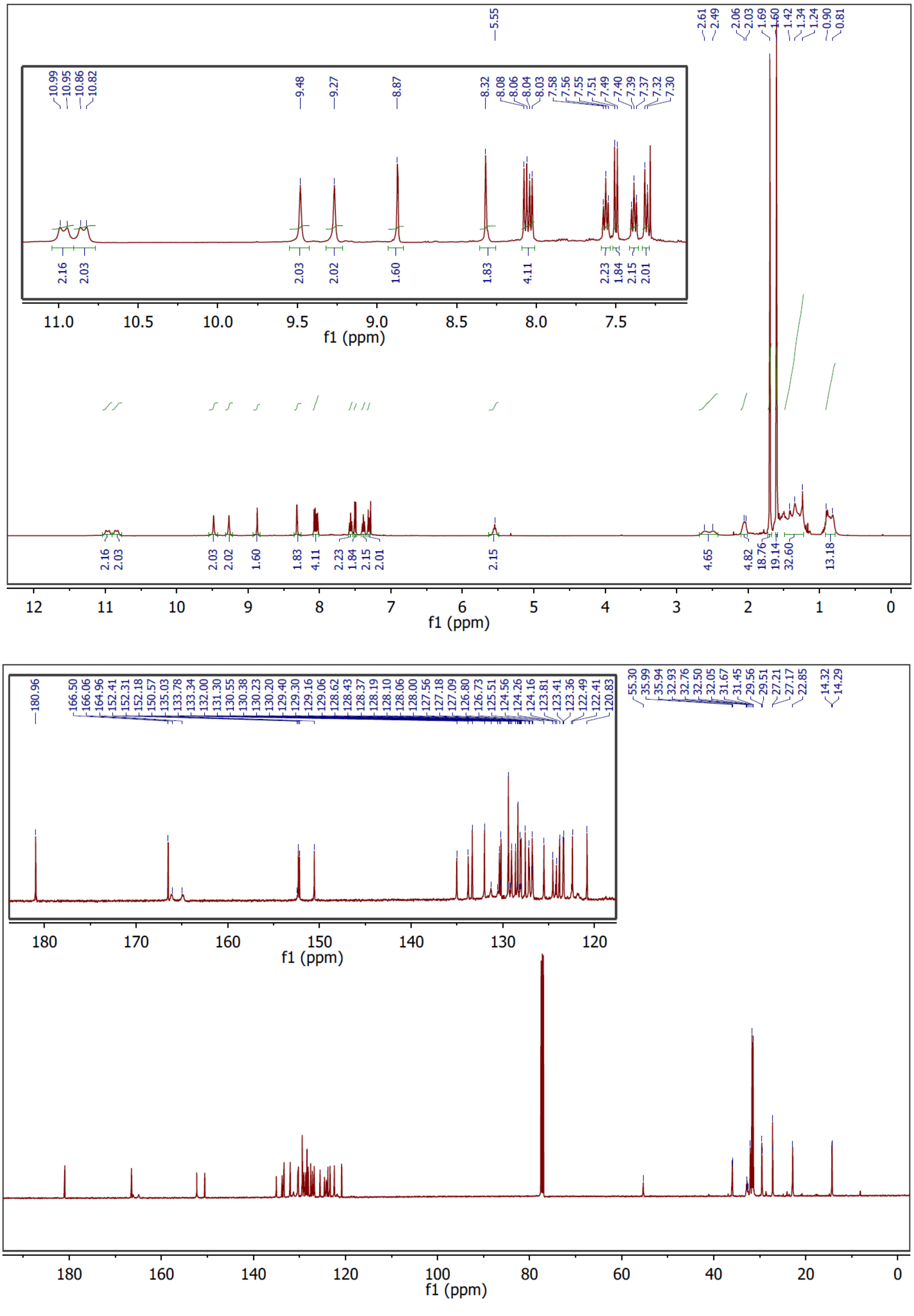

Figure S15. ^{1}H and ^{13}C NMR spectra of (***R***)-**NR-8-Q**.

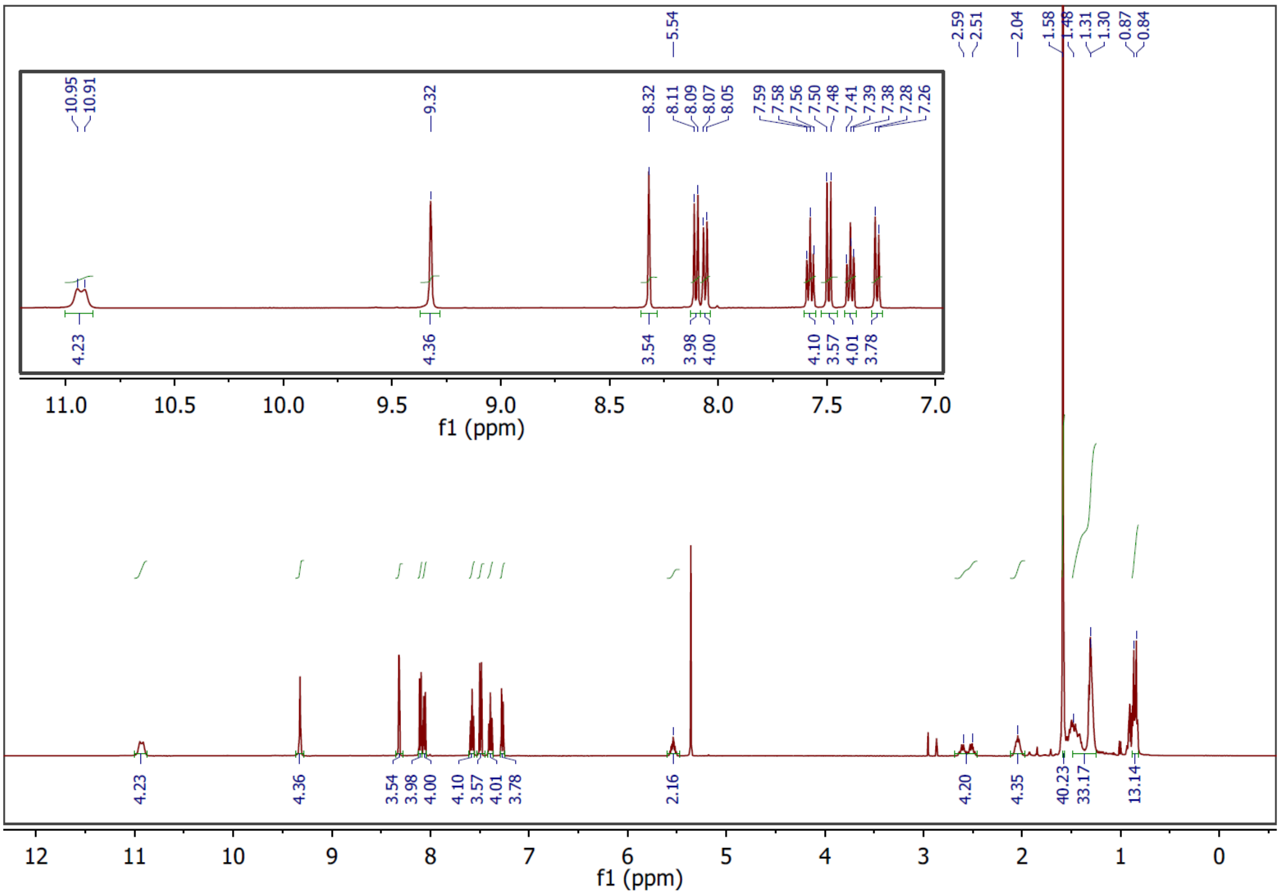

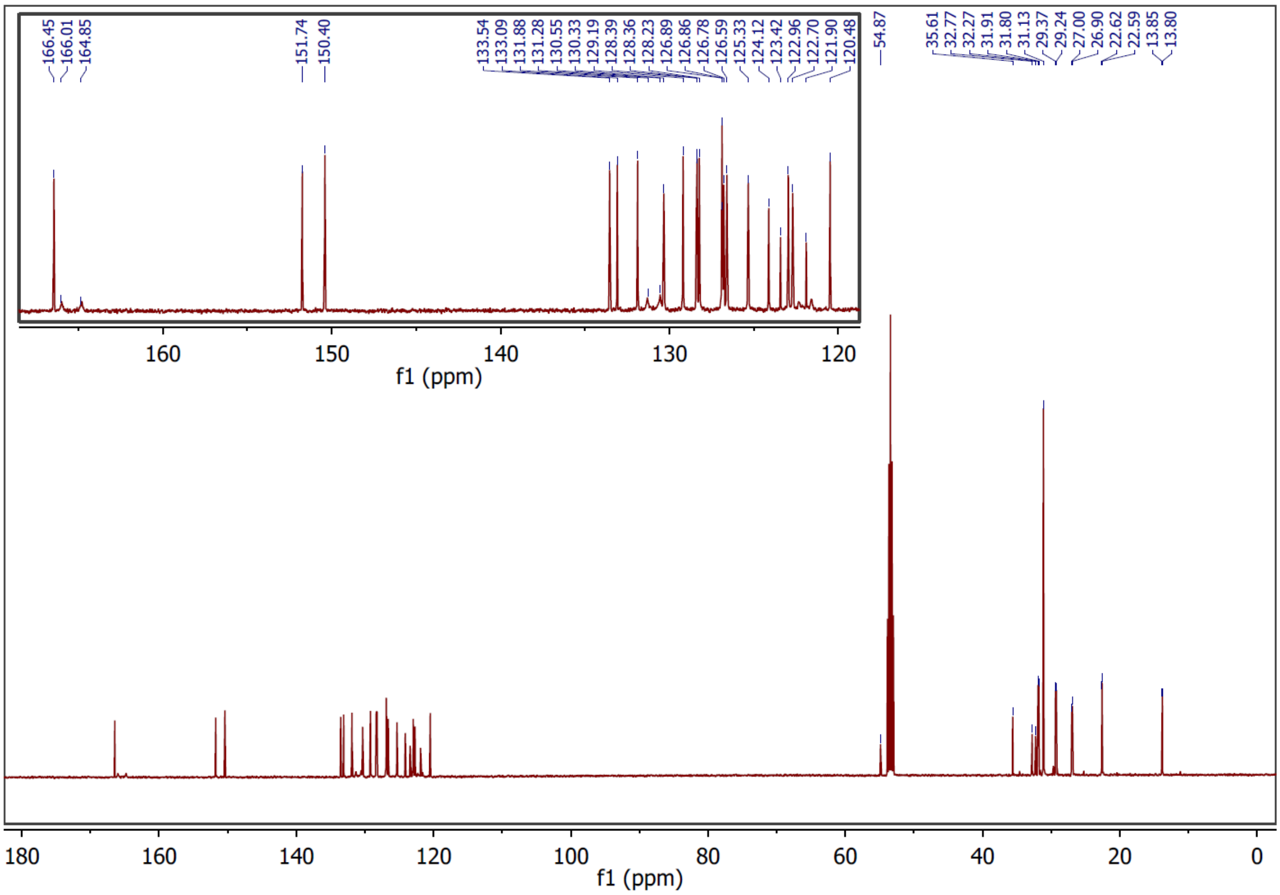

Figure S16. ^{1}H and ^{13}C NMR spectra of **(*R*,*S*)-NR-9**.

References

1. Dubey, R. K.; Melle-Franco, M.; Mateo-Alonso, A., Twisted Molecular Nanoribbons with up to 53 Linearly-Fused Rings. *J. Am. Chem. Soc.* **2021,** *143* (17), 6593-6600.
2. Magde, D.; Brannon, J. H.; Cremers, T. L.; Olmsted, J., Absolute luminescence yield of cresyl violet. A standard for the red. *J. Phys. Chem.* **1979,** *83* (6), 696-699.
3. Grimme, S., Exploration of Chemical Compound, Conformer, and Reaction Space with Meta-Dynamics Simulations Based on Tight-Binding Quantum Chemical Calculations. *J. Chem. Theory Comput.* **2019,** *15* (5), 2847-2862.
4. Bannwarth, C.; Ehlert, S.; Grimme, S., GFN2-xTB—An Accurate and Broadly Parametrized Self-Consistent Tight-Binding Quantum Chemical Method with Multipole Electrostatics and Density-Dependent Dispersion Contributions. *J. Chem. Theory Comput.* **2019,** *15* (3), 1652-1671.
5. Riplinger, C.; Sandhoefer, B.; Hansen, A.; Neese, F., Natural triple excitations in local coupled cluster calculations with pair natural orbitals. *J. Chem. Phys.* **2013,** *139* (13), 134101.
6. Frisch, M. J.; Trucks, G. W.; Schlegel, H. B.; Scuseria, G. E.; Robb, M. A.; Cheeseman, J. R.; Scalmani, G.; Barone, V.; Petersson, G. A.; Nakatsuji, H.; Li, X.; Caricato, M.; Marenich, A. V.; Bloino, J.; Janesko, B. G.; Gomperts, R.; Mennucci, B.; Hratchian, H. P.; Ortiz, J. V.; Izmaylov, A. F.; Sonnenberg, J. L.; Williams; Ding, F.; Lipparini, F.; Egidi, F.; Goings, J.; Peng, B.; Petrone, A.; Henderson, T.; Ranasinghe, D.; Zakrzewski, V. G.; Gao, J.; Rega, N.; Zheng, G.; Liang, W.; Hada, M.; Ehara, M.; Toyota, K.; Fukuda, R.; Hasegawa, J.; Ishida, M.; Nakajima, T.; Honda, Y.; Kitao, O.; Nakai, H.; Vreven, T.; Throssell, K.; Montgomery Jr., J. A.; Peralta, J. E.; Ogliaro, F.; Bearpark, M. J.; Heyd, J. J.; Brothers, E. N.; Kudin, K. N.; Staroverov, V. N.; Keith, T. A.; Kobayashi, R.; Normand, J.; Raghavachari, K.; Rendell, A. P.; Burant, J. C.; Iyengar, S. S.; Tomasi, J.; Cossi, M.; Millam, J. M.; Klene, M.; Adamo, C.; Cammi, R.; Ochterski, J. W.; Martin, R. L.; Morokuma, K.; Farkas, O.; Foresman, J. B.; Fox, D. J. *Gaussian 09 Rev. D.01*, Wallingford, CT, 2016.
7. Neese, F.; Wennmohs, F.; Becker, U.; Riplinger, C., The ORCA quantum chemistry program package. *J. Chem. Phys.* **2020,** *152* (22), 224108.
8. Jmol: an open-source Java viewer for chemical structures in 3D. http://www.jmol.org/
